# Interfacial Area Transport Equation for Bubble Coalescence and Breakup: Developments and Comparisons

**DOI:** 10.3390/e23091106

**Published:** 2021-08-25

**Authors:** Huiting Chen, Shiyu Wei, Weitian Ding, Han Wei, Liang Li, Henrik Saxén, Hongming Long, Yaowei Yu

**Affiliations:** 1 State Key Laboratory of Advanced Special Steel, Shanghai Key Laboratory of Advanced Ferrometallurgy, School of Materials Science and Engineering, Shanghai University, Shanghai 200444, China; huitingchen@shu.edu.cn (H.C.); wsywwd@shu.edu.cn (S.W.); dingweitian@shu.edu.cn (W.D.); weihan@shu.edu.cn (H.W.); 2 State Key Laboratory of Vanadium and Titanium Comprehensive Utilization, Pangang Group Research Institute Co. Ltd., Panzhihua 617000, China; liliang990@aliyun.com; 3 Thermal and Flow Engineering Laboratory, Department of Chemical Engineering, Åbo Akademi University, Biskopsgatan 8, FI-20500 Åbo, Finland; henrik.saxen@abo.fi; 4 Anhui Province Key Laboratory of Metallurgical Engineering and Resources Recycling, Anhui University of Technology, Maanshan 243000, China; yaflhm@126.com

**Keywords:** bubble coalescence and breakup, interfacial area transport equation, bubble interaction mechanisms, flow pattern transition

## Abstract

Bubble coalescence and breakup play important roles in physical-chemical processes and bubbles are treated in two groups in the interfacial area transport equation (IATE). This paper presents a review of IATE for bubble coalescence and breakup to model five bubble interaction mechanisms: bubble coalescence due to random collision, bubble coalescence due to wake entrainment, bubble breakup due to turbulent impact, bubble breakup due to shearing-off, and bubble breakup due to surface instability. In bubble coalescence, bubble size, velocity and collision frequency are dominant. In bubble breakup, the influence of viscous shear, shearing-off, and surface instability are neglected, and their corresponding theory and modelling are rare in the literature. Furthermore, combining turbulent kinetic energy and inertial force together is the best choice for the bubble breakup criterion. The reviewed one-group constitutive models include the one developed by Wu et al., Ishii and Kim, Hibiki and Ishii, Yao and Morel, and Nguyen et al. To extend the IATE prediction capability beyond bubbly flow, two-group IATE is needed and its performance is strongly dependent on the channel size and geometry. Therefore, constitutive models for two-group IATE in a three-type channel (i.e., narrow confined channel, round pipe and relatively larger pipe) are summarized. Although great progress in extending the IATE beyond churn-turbulent flow to churn-annual flow was made, there are still some issues in their modelling and experiments due to the highly distorted interface measurement. Regarded as the challenges to be addressed in the further study, some limitations of IATE general applicability and the directions for future development are highlighted.

## 1. Introduction

Gas-liquid two-phase flow plays a critical role in natural and industrial systems, which exists in air bubbles in the ocean [1], steam in the water of an electric power plant [2], carbon dioxide in methylamine of the chemical engineering [3], and oxygen gas in the hot metal of steelmaking [4]. The gas-liquid flow generates the deformation in two phases and leads to a constant change of the interphase and the medium distribution, which greatly affects the flow, heat, and mass transfer characteristics. Different flow rates, pressure, channel layout, and channel geometry can result in a different distribution of phase interface in gas-liquid two-phase flow, that is, different flow structure modes are formed. Therefore, depending on the gas, flow properties, and container geometry, the gas-liquid flow patterns are classified: stratified (horizontal channels), slug, churn, annular, and bubbly flow.

Under certain conditions, the fluctuation of a gas-liquid interface is unstable in stratified flow, leading to a hydrodynamic slug. Slug flow is characterized by alternating slugs of liquid and large gas bubbles. In annular flow, the liquid flows around the inner circumference of the pipe as a film, while gas flows in the center of the pipe. The film becomes thinner and more liquid is carried by the gas as a mist when the gas velocity increases in annular flow. Dispersed bubbly flow is the opposite of annular flow. Bubbly flow is distributed in the liquid phase as variable size, deformable bubbles moving upward, and undergo a transition to slug flow when the gas superficial velocity increases due to the corresponding rise in the volume fraction and the coalescence of bubbles. It is readily acknowledged that the formation and motion of bubbles and interaction mechanisms of bubbles are very complicated and the gas-liquid interfacial area is the deciding factor in controlling mass and heat transfer in gas-liquid two-phase flow. As the basis of bubble motion, bubble coalescence and breakup are often regarded as a game, just like playing soap bubbles at early ages, but have a wide range of applications in natural and industrial systems. In the mass flux between the sea surface and the atmosphere, bubble breakup is considered a source of aerosol droplets in the air [5]. In metallurgical processes, gas bubbles are injected at the bottom of bulk liquid metal to mix and homogenize the metal, where the importance of bubble coalescence and breakup cannot be emphasized enough [6]. In transdermal drug delivery, the bubble with controlled size can elevate the efficiency of drug delivery and reduce medical waste [7]. As for the biotechnology industry, bubbles that rise to the surface of cell suspension can damage cells, when they break up [8]. In the food industry, a large of bubbles and foams are needed to form cavities and produce low-density fillers in the fields like adhesives. In the coating industry, the defoamer which prevents bubble formation can improve the quality of paint film. Therefore, the principle of bubble coalescence and breakup is an important problem to be solved urgently in industry, and bubble interactions are studied experimentally and calculated by scientists and engineers in various industries in the past and in the future.

As the determination of flow patterns in a pipe is the precondition in the computation, many approaches were developed for flow pattern maps and flow transition criterion according to own term, which can be used to predict the specific bubble behaviors with a given set of local variables. In the beginning, 1D system codes are frequently used for the description of two-phase flow, which is based on the empirical work and can predict the gas-liquid flow in the case of the given volume flow rates of liquid and gas. However, these flow patterns can neither predict the flow pattern changes along the flow path nor the flow structures with time and space. Then, a 3D computational fluid dynamics (CFD) program has been tried to solve the above issue by combining it with additional transport equations for fluid particle number density or its corresponding parameters in the advanced two-phase flow model. On account of generous theoretical and simulation analysis, the definition of bubble interaction mechanisms is very important to obtain accurate and reliable two-phase flow phenomena by CFD simulation. A better method than algebraic approximation is to directly use transport equation to simulate the interfacial concentration, especially for the 3D of two-phase flow. Therefore, the interfacial area transport equation (IATE) developed from the population balance equation is one of the prospective ways to dynamically describe the temporal and spatial evolutions. A major advantage of IATE is that the evolutions of the interfacial area concentration (IAC) can be modeled without flow patterns.

The paper presents a review of constitutive models for gas-liquid two-phase flow in the publications, especially for fundamental issues. The transition of flow patterns of bubble coalescence and breakup are introduced in Section 2, transport equations of bubbles are provided in Section 3. Then, bubble interaction mechanisms and comparisons of constitutive models for bubble coalescence and breakup are generalized in Section 4 and Section 5, respectively. In Section 6, conclusions and future developments are highlighted.

## 2. Transition of Bubbly to Churn-Turbulent Flows

IAC is an essential parameter of the phase interaction terms in the two-phase model, and its dynamic prediction has been realized by IATE [9]. It is consistent with the change mechanism of the interfacial area and able to predict the patterns without static flow. So, discontinuity caused by the transition between flow patterns can be avoided. In order to predict IAC, the physical mechanisms of bubbly to churn-turbulent flows should be studied first.

### 2.1. Bubbly to Slug Transition

**Figure 1:**
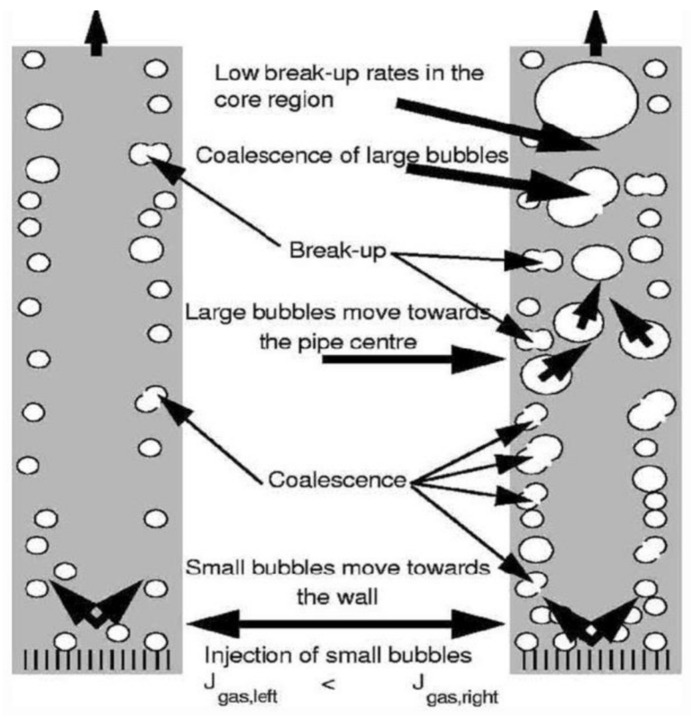
Stable bubbly flow (**left**) and transition to slug flow (**right**) [10].

In bubbly flow, fluid turbulence is an important cause of bubble interactions. Transfer motion and interaction of bubbles affect the flowing path, and the flow evolution is determined by the interactions of bubble migration, coalescence, and breakup as shown in Figure 1 [10]. In Figure 1 (left), a stable bubbly flow is observed far away from the transition region to slug flow and bubbles are nearly spherical. Upward bubbly flow has the wall-peaked void profile, while downward bubbly flow concentrates some small bubbles at the pipe center. Coalescence and breakup occur among bubbles with approximately the same diameter. In Figure 1 (right), the bubbly flow changes to slug flow obviously, where there are both nearly spherical bubbles and elliptical or cap bubbles. In such a case, larger bubbles in the form of ellipses or cap bubbles concentrate in the central region. Finally, two or more cap bubbles merge into a bigger bubble. If the bubble is sufficiently large, it will fill most of the cross-section of the pipe, and slug flow may result. For bubbles with different sizes have significantly different decomposition volume fraction and turbulent energy dissipation rates, bubble coalescence, and breakup should be considered locally.

As the above discussion, the transition from dispersed bubbly to slug flow observed at low gas flow rates requires a coalescence process, where discrete bubbles with a diameter are closed to the pipe form a larger vapor space. As the gas rate increases, so does the amount of gas in the pipe. In such a case, tighter bubbles lead to an increase in the coalescence rate. Meanwhile, turbulence fluctuation can lead to the breakup of large bubbles in the coalescence process, as the liquid rate increases. If the breakup rate is high enough to avoid recoalescence, dispersed bubbles can be maintained. Therefore, a critical size of 15 mm for air-water is observed, bubbles deform and move randomly along the zigzag path [11]. They collide and coalesce, forming larger-cap bubbles similar to Taylor bubbles but with a diameter smaller than the pipe. In a high gas flow rate and a low liquid flow rate, the bubble packing intensifies. The value of volume fraction at which the transition to slug flow discussed above is about 0.25 to 0.3 [11].

### 2.2. Slug to Churn Transition

If small bubbles coalesce to form Taylor bubbles, a slug flow can form from the bubbly flow. If two serial Taylor bubbles are too small to be stable, a churn flow occurs. As Taitel et al. mentioned [11], churn flow is a developing length region for slug flow in essence. If a position along the pipe length is shorter than the developing length, churn or slug flow can be observed. On the contrary, only the slug flow exists.

### 2.3. Slug/Churn to Anular Flow Transition

For a high gas flow rate, the flow goes annular and the liquid film flows upward along the wall. The gas flows in the center, carrying droplets. The film flows uphill against gravity because the rapid-moving gas core exerts a force on it. This film has a wavy interface, which can be broken easily and enter the gas core in the form of droplets. In fact, when the gas flow rate is low, droplets will fall back and accumulate, resulting in churn or slug flow.

## 3. Interfacial Area Transport Equation (IATE) for Bubble Coalescence and Breakup

**Figure 2:**
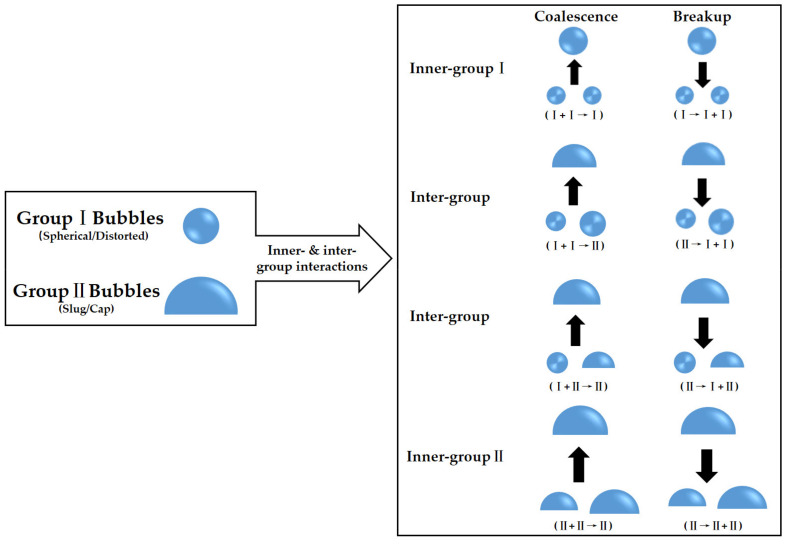
Classification of possible interactions of two-group bubbles.

As mentioned in Section 2, bubble coalescence and breakup should be considered locally for various bubbles in bubbly, slug, churn, and annular flow patterns. IATE can handle these bubbles in two groups: the spherical/distorted bubble group and the cap/slug bubble group, leading to four-type interactions as shown in Figure 2.

**Figure 3:**
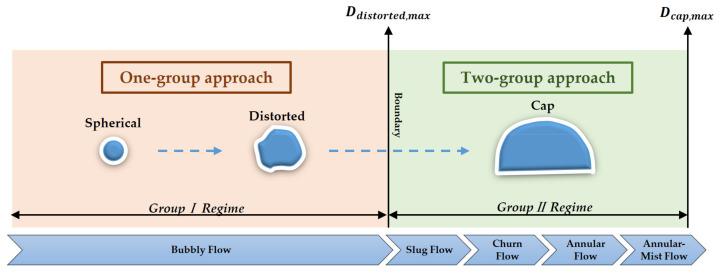
Schematic diagram of two-group approach.

Two kinds of IATE for all bubbles are possible and needed to cover the flow patterns including bubbly, slug, churn, and annular flow as seen in Figure 3. Group I bubbles (spherical and distorted bubbles) in bubbly flow are treated in one-group IATE, while others (cap-shaped bubbles) are managed into two-group IATE in slug, churn, and annular flow. If there are not Group II bubbles, two-group IATE can be simplified to one-group IATE [12]. In addition, the main challenge of the two-group IATE comes from the complex modeling of inner- and inter-group interactions (as seen in Figure 2), and two-group bubbles provide slight contributions at low volume fraction which may be ignored for practical modeling [13]. Constitutive models of one-group IATE which can discuss the dynamic characteristics of interfacial area enough is our main focus.

Based on Boltzmann transport equation, an IATE was formulated by Kocamustafaogullari and Ishii [9] as:

(1) $$\frac{\partial f}{\partial t}+\nabla \cdot \left(f\vec{v}\right)+\frac{\partial \left(f\frac{dV}{dt}\right)}{\partial V}=\sum_{j}S_{j}+S_{ph},$$

where *f*(*V*, $\vec{x}$, *t*) is the bubble number density distribution function. The right-hand side (RHS) of this equation is the source and sink terms. The first term $S_{j}$ represents the net rate of change in the number density distribution function due to the bubble breakup and coalescence processes, while $S_{ph}$ is the bubble number sink or source rate due to phase change.

Even though the Boltzmann transport equation is much more macroscopic, a better version is the bubble number density transport equation. As the IAC of bubbles is related to the bubble number and size [14], the IATE can be derived from Equation (1). Applying the Leibnitz integration rule, the bubble number density transport equation can be written as:

(2) $$\frac{\partial n}{\partial t}+\nabla \cdot \left(n{\vec{v}}_{pm}\right)=\sum_{j}R_{j}+R_{ph},$$

where ${\vec{v}}_{pm}$, the average local particle velocity, *n* is the number of particles of all sizes per unit mixture volume and $R_{j}$ are the source and sink rates.

### 3.1. One-group IATE for Bubble Coalescence and Breakup

Similar to the above, Ishii and Hibiki [14] developed their IATE as follows.

(3) $$\frac{\partial \alpha_{i}}{\partial t}+\nabla \cdot \left(\alpha_{i}{\vec{v}}_{i}\right)-\left(\frac{1}{V}\frac{dV}{dt}\right)\int_{V_{min}}^{V_{max}}\mathrm{fVd}A_{i}=\int_{V_{min}}^{V_{max}}\sum_{j}(S_{j}+S_{ph})A_{i}dV,$$

where $\alpha_{i}$ is the average interfacial area density of all bubbles of volume $V_{min}$ and $V_{max}$ and ${\vec{v}}_{i}$ is the interfacial velocity.

For the expression of volume source one obtained:

(4) $$\frac{\partial \alpha_{i}}{\partial t}+\nabla \cdot \left(\alpha_{i}{\vec{v}}_{i}\right)-\frac{2}{3}\left(\frac{\alpha_{i}}{\alpha }\right)\left[\frac{\partial \alpha }{\partial t}+\nabla \cdot \left(\alpha {\vec{v}}_{g}\right)-\zeta_{ph}\right]=\int_{V_{min}}^{V_{max}}\sum_{j}(S_{j}+S_{ph})A_{i}dV,$$

The third term on the left-hand side (LHS) is the change of interfacial area density due to bubble volume change, the source term expresses $\int_{V_{min}}^{V_{max}}\sum_{j}S_{j}dV=\sum_{j}R_{j}$ (in the bubble number density transport equation) and the sink term is $\int_{V_{min}}^{V_{max}}\sum_{j}S_{j}A_{i}dV=\sum_{j}\Phi_{j}$ (in the interfacial area density transport equation), where $\Phi_{ph}=\pi D_{bc}^{2}R_{ph}$, and $D_{bc}$ is the bubble critical size due to phase change.

**Figure 4:**
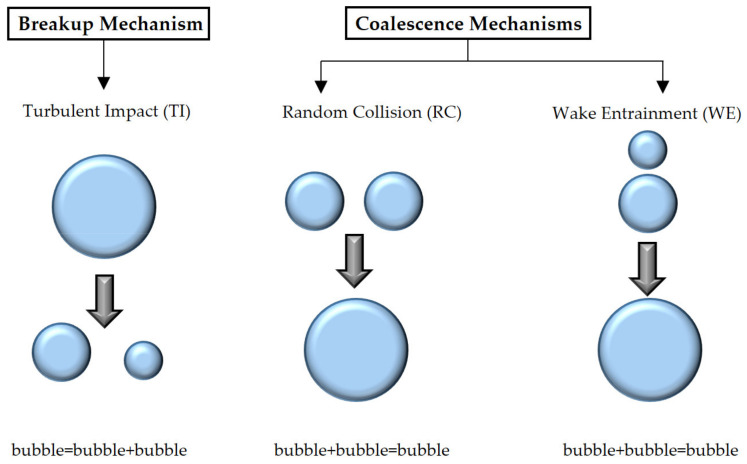
Spherical bubble interaction mechanism in bubbly flow.

Bubble-bubble and bubbles-turbulence interactions lead to the temporal and spatial evolution of gas-liquid two-phase flow, which has aroused extensive attention. Models of interaction area transfer proposed by Wu et al. [13,15], Ishii and Kim [16], Hibiki and Ishii [17], Yao and Morel [18], and Nguyen et al. [19] were used widely. Of these numerous studies pertaining to bubble coalescence and breakup for bubbly flow, the main phenomenon and mechanism have been summarized: (a) bubble breakup due to turbulent impact (TI); (b) bubble coalescence due to random collision (RC); (c) bubble coalescence due to wake entrainment (WE). Major bubble interaction mechanisms in bubbly flow are shown in Figure 4. With bubble Reynolds number increase, the shape of bubbles, mechanism, and constitutive equations changes.

Taking three bubble interactions above into consideration, Wu et al. [15], Ishii et al. [20], and Kim [21], formulated the one-group IATE for bubbly flow. In their studies, a one-group IATE is developed:

(5) $$\frac{\partial \alpha_{i}}{\partial t}+\nabla \cdot \left(\alpha_{i}{\vec{v}}_{i}\right)=\frac{2}{3}\left(\frac{\alpha_{i}}{\alpha }\right)\left[\frac{\partial \alpha }{\partial t}+\nabla \cdot \left(\alpha {\vec{v}}_{g}\right)-\zeta_{ph}\right]+\frac{1}{3\psi }{\left(\frac{\alpha }{\alpha_{i}}\right)}^{2}\left(R_{TI}-R_{RC}-R_{WE}\right)+\pi D_{bc}^{2}R_{ph},$$

where $R_{TI}$, $R_{RC}$ and $R_{WE}$ are the bubble number source/sink rate due to turbulent impact, random collision, wake entrainment, respectively. The LHS in Equation (5) represents the time-dependent and the convective rate of change of the interfacial area density. In the RHS, the first term represents bubble expansion or compression due to pressure effects, the second term is the rate of change due to bubble interaction mechanisms and, the third, due to phase change.

The mechanism of the IATE is likely to depend on the bubble mixing length, turbulence intensity, and volume fraction. Therefore, the evolution of the number density distribution is described by the destruction and creation of the population due to bubble coalescence and breakup. In order to understand the mechanisms of bubble transport and the transient change of the flow patterns, the mechanisms and dynamics of coalescence and breakup must be studied systemically as summarized in Section 4.

### 3.2. Two-Group IATE for Bubble Coalescence and Breakup

A wide variety of bubbles sizes and shapes result in various bubble-turbulence and bubble-bubble interaction mechanisms. Considering bubbles with various sizes and shapes under a given set of gas-liquid two-phase flow conditions, an IATE that can manage the transport characteristics of various bubbles is necessary. As shown in Figure 3, Group I bubbles only exist in the spherical and distorted bubble regime, while Group II bubbles exist in the cap bubble regime. The boundary of two regimes is given by Ishii and Zuber [22] as the following Equations (6) and (7), respectively:

(6) $$D_{distorted,max}=4\sqrt{\frac{\sigma }{g\Delta \rho }},$$

(7) $$D_{cap,max}=40\sqrt{\frac{\sigma }{g\Delta \rho }},$$

where $D_{distorted,max}$ is the maximum critical size of distorted bubbles in Group I, $D_{cap,max}$ is the maximum stable bubble size limit, σ is the surface tension, and Δρ is the density difference.

Averaging the volume range of each group in Group II, a two-group IATE was obtained by Ishii and Kim [12].

(8) $$\frac{\partial \alpha_{i1}}{\partial t}+\nabla \cdot \left(\alpha_{i1}{\vec{v}}_{i1}\right)=\frac{2}{3}\left(\frac{\alpha_{i1}}{\alpha_{1}}\right)\left[\frac{\partial \alpha_{1}}{\partial t}+\nabla \cdot \left(\alpha_{1}{\vec{v}}_{g1}\right)-\zeta_{ph}\right]+\int_{V_{min}}^{V_{cr}}\sum_{j}(S_{j}+S_{ph})A_{i}dV-\chi {\left(\frac{D_{sc}}{D_{sm1}}\right)}^{2}\frac{\alpha_{i1}}{\alpha_{1}}\left[\frac{\partial \alpha_{1}}{\partial t}+\nabla \cdot \left(\alpha_{1}{\vec{v}}_{g1}\right)-\zeta_{ph}\right],$$

(9) $$\frac{\partial \alpha_{i2}}{\partial t}+\nabla \cdot \left(\alpha_{i2}{\vec{v}}_{i2}\right)=\frac{2}{3}\left(\frac{\alpha_{i2}}{\alpha_{2}}\right)\left[\frac{\partial \alpha_{2}}{\partial t}+\nabla \cdot \left(\alpha_{2}{\vec{v}}_{g2}\right)\right]+\int_{V_{cr}}^{V_{max}}\sum_{j}S_{j}A_{i}dV+\chi {\left(\frac{D_{sc}}{D_{sm1}}\right)}^{2}\frac{\alpha_{i1}}{\alpha_{1}}\left[\frac{\partial \alpha_{1}}{\partial t}+\nabla \cdot \left(\alpha_{1}{\vec{v}}_{g1}\right)-\zeta_{ph}\right],$$

where χ is the inter-group transfer coefficient, $V_{cr}$ is the critical bubble volume, $D_{sc}$ and $D_{sm1}$ are the surface equivalent diameter and Sauter mean diameter, and subscripts 1 and 2 stand for Group I andII, respectively. The LHS of Equations (8) and (9) are the gross changing rate for IAC of each group and the terms in the RHS are the changing rate for IAC due to particle volume change, phase change, and intergroup transfer due to particle volume change and various particle interactions.

In the latest study proposed by Worosz [23], two-group IATE was statistically obtained as follows.

(10) $$\frac{\partial \alpha_{i1}}{\partial t}+\nabla \cdot \left(\alpha_{i1}{\vec{v}}_{i1}\right)=\frac{2}{3}\left(\frac{\alpha_{i1}}{\alpha_{1}}\right)\left[\frac{\Gamma_{g1,i}}{\rho_{g}}-\frac{\alpha_{1}}{\rho_{g}}\frac{D_{g1}\rho_{g}}{Dt}\right]-\chi \frac{A_{icr}}{{\bar{A}}_{i1}}\left(\frac{\alpha_{i1}}{\alpha_{1}}\right)\left[\frac{\Gamma_{g1,i}}{\rho_{g}}-\frac{\alpha_{1}}{\rho_{g}}\frac{D_{g1}\rho_{g}}{Dt}\right]+\sum_{j}\Phi_{j,1}+\Phi_{nuc,1},$$

(11) $$\frac{\partial \alpha_{i2}}{\partial t}+\nabla \cdot \left(\alpha_{i2}{\vec{v}}_{i2}\right)=\frac{2}{3}\left(\frac{\alpha_{i2}}{\alpha_{2}}\right)\left[\frac{\Gamma_{g2,i}}{\rho_{g}}-\frac{\alpha_{2}}{\rho_{g}}\frac{D_{g2}\rho_{g}}{Dt}\right]-\chi \frac{A_{icr}}{{\bar{A}}_{i1}}\left(\frac{\alpha_{i2}}{\alpha_{2}}\right)\left[\frac{\Gamma_{g1,i}}{\rho_{g}}-\frac{\alpha_{1}}{\rho_{g}}\frac{D_{g1}\rho_{g}}{Dt}\right]+\sum_{j}\Phi_{j,2}+\Phi_{nuc,2},$$

where $\Phi_{nuc}$ is interfacial area concentration source/sink rate due to nucleation. Therefore, two sets of equations are used to describe the generation and destruction rates of bubble number density, volume fraction, and IAC for two-group bubbles due to expansion and compression, coalescence and disintegration, and phase change.

The one-group IATE proposed by Worosz is consistent with the one by Ishii and Kim [12], but a difference in the view on two-group IATE (Equations (10) and (11)). In the two-group IATE of Ishii and Kim (Equations (8) and (9)), the effects of the intergroup transfer due to particle interactions and particles expansion across the group boundary are considered into particle volume changing rate and they are double-calculated.

For the sink term and source term concerning the bubble coalescence and breakup mechanism are the main components of IATE, the constitutive models of bubble coalescence, and breakup which are usually established from experimental data based on various flow conditions is largely dependent on the performance of IATE. Thus, there are a large number of bubble coalescence and breakup models which are usually supported by the experimental data. However, recent findings [23,24] indicated that the performance of IATE in transition flow is not satisfactory. There are still some issues and difficulties: the establishment of bubble behavior turbulence model, bubble coupling of mechanisms, the lack of reliable experimental data, and the simultaneous occurrence of coalescence and breakup. Therefore, an evaluation of existing bubble coalescence and breakup constitutive models in the literature is needed as a basis for further developments, which is reviewed in Section 5.

## 4. Identification of Bubble Coalescence and Breakup Interaction Mechanisms

**Figure 5:**
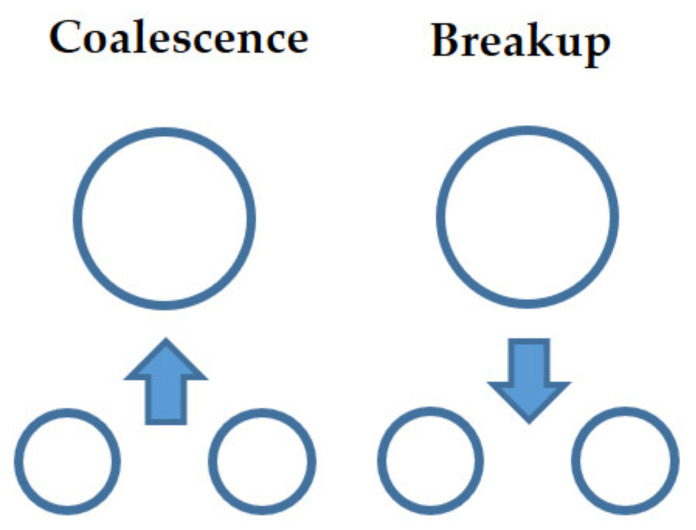
Schematic diagram of bubble coalescence and breakup.

As previously mentioned in Section 3, most coalescence and breakup mechanisms are based on the assumption of the interactions among spherical bubbles. Bubble interaction and transfer mechanisms depend on bubble shapes. Due to the wide range of bubble shapes and sizes, a set of transport equations describing bubble transport in a two-phase flow is needed. Fully understanding the dynamics of a single bubble is the initial form of multi bubble system modeling and is a fundamental issue of bubbly flow. Therefore, most publication papers study the behavior of a single isolated bubble and the transfer phenomenon of dispersed bubbles in bubbly flow. The process is shown schematically in Figure 5.

### 4.1. Bubble Coalescence Mechanisms

Bubbles collide with each other constantly in involving gas-liquid dispersion. The phenomenon of coalescence entails three stages: initial contact between bubbles, controlled essentially by the hydrodynamics of the liquid, which leads to a film of the thickness of a few microns separating the two bubbles. The second step is the thinning to a few Angstroms, of which the rate is decided by the hydrodynamics of thin films. The final step is the rupturing of this film resulting in the coalescence. Obviously, the rate of bubble drainage and thinning in the second stage determines whether the bubbles coalesce or not. If the drainage time to rupture is longer than the contact time, the two bubbles will separate rather than coalesce. The final step, or the rupturing step, is usually faster than the other two stages.

**Figure 6:**
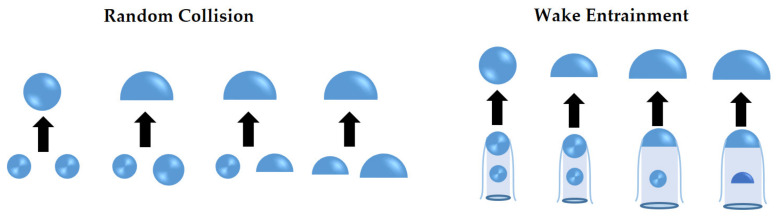
Schematic diagram of bubble coalescence mechanisms.

The large spherical cap bubble with a strong wake region may lead to the collision and coalescence of other bubbles when others enter the wake region and accelerate. Bubble coalescence depends on bubble size, velocity, and collision frequency. Two main coalescence mechanisms have been identified: bubble coalescence due to random collision (RC) and bubble coalescence due to wake entrainment (WE) as shown in Figure 6.

### 4.2. Bubble Breakup Mechanisms

**Figure 7:**
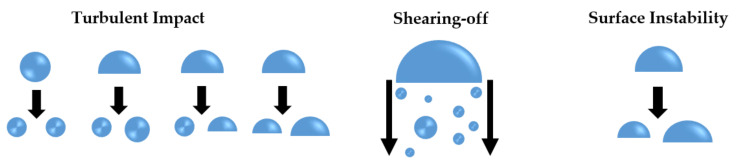
Schematic diagram of bubble breakup mechanisms.

A bubble disintegrates into two or more new bubbles in the breakup process, and the interfacial area increases and so does the interfacial transfer between phases. Group I bubbles (spherical/distorted bubbles) break up due to the turbulent impact (TI) of the eddies against the bubble as shown in the left of Figure 7. As for Group II bubbles (caps or slugs), smaller bubbles shear off from them with a low viscosity fluid as shown in the intermedia of Figure 7, called shearing-off (SO). When the properties of the bubble surface are unstable, smaller bubbles are formed from the unstable bubbles as shown in the right of Figure 7. This breakup process is due to surface instability (SI).

#### 4.2.1. Breakup due to Turbulent Impact

When the amplitude of the oscillation is close to the amplitude needed to destabilize the surface of the bubble, it begins to deform and stretch in a direction. This leads to the further contraction of the neck and finally breaks into two or more bubbles. Furthermore, bubble breakup is attributed mainly to pressure fluctuations along its surface and by bubble-eddy collisions in the moving fluid or turbulent liquid. In the view of the force required to cause a breakup, the disintegration process can be expressed as the balance between dynamic pressure and its surface stress. Hence, the criterions in the literature are summarized due to different conditions and various bubble properties (see Table 1). There are three categories for the breakup: turbulent kinetic energy, fluid velocity, or inertial force hitting on the bubbles. As turbulent kinetic energy is the function of fluid velocity, (a) and (b) in Table 1 have a similar effect on breakup behaviors. If there is no turbulent flow in fluid and the bubble with a low velocity is broken up by an object, behaviors of the bubble can be explained by the two criterions above. Therefore, d) is a solid option. In brief, combining turbulent kinetic energy (velocity) and inertial force together was proposed by Wang et al. [25] and Zhao and Ge [26] is the best choice for the bubble breakup criterion.

#### 4.2.2. Breakup due to Shearing-Off

With the increasing bubble size, breakup mechanisms become much more complicated owing to additional phenomena such as shearing-off and interfacial instability, both of which are induced by a velocity difference across the interface. During the shearing-off process, that many small bubbles are sheared off from a large bubble is called erosive breakage. When the relative velocity is high enough to make the bubble skirt unstable, the bubble skirt can fall off from the large one with the generation of small bubbles at the edge. However, experiments and theoretical studies have revealed that the interfacial viscous shear force can be neglected due to the low viscosity of water in the air-water flow, in which the shearing-off process is caused by the gas distribution inside the cap bubble [40]. In a word, the shearing-off is determined by the balance between the viscous shear force and the surface tension at skirts of the cap/slug bubbles in highly viscous flows

#### 4.2.3. Breakup due to Viscous Shear Forces

When the fluid has a high viscosity, viscous shear forces in the continuous phase lead to a velocity gradient near the interface, resulting in bubble deformation and final breakup. If a large part of a trailing bubble locates outside the wake region, the shear stress across the wake boundary may split the bubble due to extension, and the bubble surface is depressed and necked. In the first stage, the bubble is elongated into two lumps by a thread and then divided into two equal-sized daughter bubbles corresponding to the lumps and a series of smaller bubbles called satellites. The bubble may also break into a cylindrical thread which may break into many smaller bubbles, where it is called thorough the breakage [9,41,42]. In terms of the viscous case, the breakup mechanism is expressed as the force balance between surface tension forces and external viscous stresses, which is usually formulated as a dimensionless Capillary number.

(12) $$\mathrm{Ca}=\frac{\tau_{v}}{\tau_{s}},$$

Expressed as the ratio of viscous stress over the surface tension, it can be formulated as

(13) $$\mathrm{Ca}=\frac{\mu_{f}d\dot{\gamma }}{2\sigma },$$

If the Capillary number or bubbles size surpasses the critical value, that is, Ca ≥ ${\mathrm{Ca}}_{\mathrm{cr}}$, interfacial tension forces cannot keep the form of the bubble, leading to the breakup of two or more daughter bubbles. The critical diameter is determined by

(14) $$D_{cr}=\frac{2\sigma {\mathrm{Ca}}_{\mathrm{cr}}}{\mu_{f}\dot{\gamma }},$$

As for the case of turbulent flows, the viscous force includes the laminar shear in the bulk flow and the turbulent one $\sqrt{\frac{\varepsilon }{\nu }}$. The latter one is responsible for the collision of bubbles with the size smaller than the Kolmogorov length scale. Briefly, the breakup mechanism due to viscous shear force is expressed by the force balance between surface tension forces and external viscous stresses.

#### 4.2.4. Breakup due to Surface Instability

The mechanisms above depend on the dynamic characteristics of continuous flow. However, it is proved that even if there is no net flow in the continuous phase, the interface instability will lead to breakup. The breakup process due to SI includes two instabilities: Rayleigh–Taylor instability (RTI) and Kelvin–Helmholtz instability (KHI). RTI is common in natural and industrial processes. It describes the formation of irregularities along the gas-liquid interface, as it is accelerated in a direction perpendicular to the gas-liquid interface of bubbles. That is, when there is a density difference in two fluids, RTI occurs. KHI arises due to shear along an interface between the gas and liquid. Thus, when the density ratio is approximately 1, the breakup process is dominated by RTI.

In general, five bubble interaction mechanisms in the general two-phase flow are summarized. They are (1) RC: bubble coalescence motivated by turbulent eddies; (2) WE: bubble coalescence due to acceleration of the following bubbles in the wake of a leading bubble; (3) TI: bubble breakup due to the impact of turbulent eddies; (4) SO: small bubbles shearing-off from the rim of a large bubble; (5) SI: breakup due to surface instability at the interface. Table 2 summarizes the inner- and inter-group interactions based on the discussion above. Superscript (1) stands for Group I bubbles and (2) for Group II bubbles.

## 5. Evaluation of Frequency Models and Constitutive Models for Bubble Coalescence and Breakup

The bubble interaction mechanisms of coalescence and breakup discussed in Section 4 should be pursued as constitutive relations to solve the IATEs. In this section, several coalescence and breakup frequency expressions are reviewed, classified, and compared. Furthermore, a literature review regarding the bubble coalescence and breakup constitutive models is presented, followed by discussions and conclusions.

### 5.1. Frequency Models

#### 5.1.1. Breakup Frequency Models

**Figure 8:**
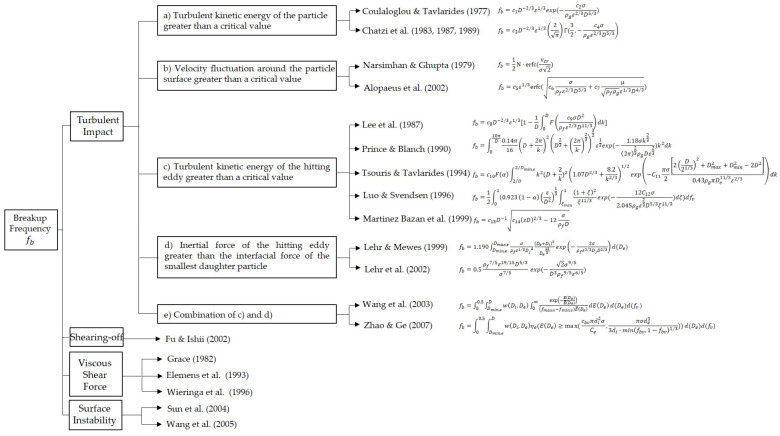
Classification of bubble breakup models.

In the past few years, great progress has been made in the analysis and modeling of the breakup process, and a large number of breakup frequency and daughter size distribution models have been proposed. In Figure 8, there are a large number of breakup frequency models, which are classified into four parts according to the discussion in Section 4.2, namely, turbulent impact, shearing-off, viscous shear force, and surface instability.

**Figure 9:**
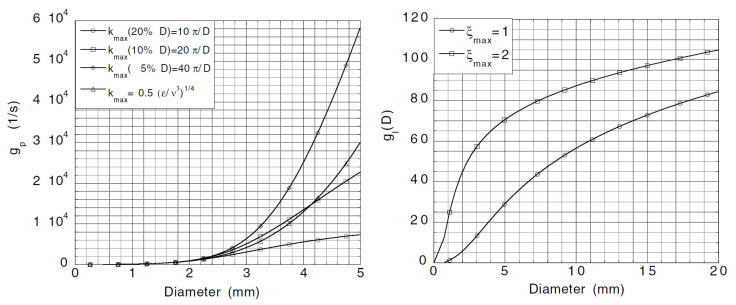
Breakup frequency calculated as Prince and Blanch model (**left**) and Luo and Svendsen model (**right**) for different values, *σ* = 0.072 N/$m^{-1}$, $\rho_{f}$ = 1000 kg/$m^{3}$ and *ε* = 1 $m^{2}$/$s^{3}$ [44].

Turbulence is the most common situation, on which breakup models are focused. Compared to the turbulent impact, the influence of viscous shear, shearing-off, and surface instability in a turbulent flow is usually neglected and the corresponding theory and modeling are rare in the literature. In the classification of turbulent impact, Prince and Blanch [34] set the upper limit of the integration as 10π/D arbitrarily, rather than the lower limit of integration. They believed that there is not enough energy to break up bubbles in the case of eddies with characteristic dimensions less than 20% of the bubble size. Finally, it is appropriate to point out here that the models by Prince and Blanch [34] and by Luo and Svendsen [36] are sensitive to the upper and lower limit of the integration, so they cannot be chosen arbitrarily. Different values of the upper integration limit and of the lower integration limit have been tested and the results are shown in Figure 9.

Most of these breakup frequency models are based on a theory fundamentally similar to the kinetic theory of gases, and the models above assume that turbulence is considered as an array of eddies with well-defined sizes and densities. After defining a collision cross-section and obtaining an eddy arrival frequency, closure parameters that change the conditions, such as integration limits, have to be set. However, it is impossible to prove the models based on the eddy concept, and it is too difficult to obtain correct and reliable data concerning the density or size of eddies that take part in interactions with bubbles.

#### 5.1.2. Coalescence Frequency Models

**Figure 10:**
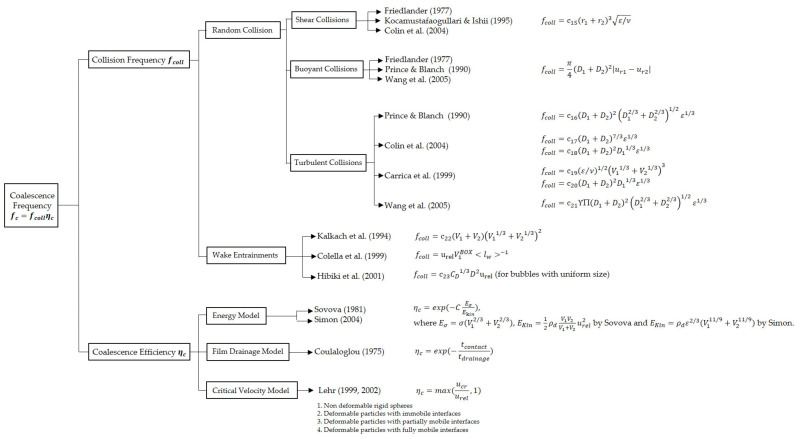
Classification of bubble breakup models.

Coalescence is considered to be more complex than breakup, for the interactions of bubbles and between bubbles and surrounding liquid are involved, once they are gathered together by outflow or external force. The coalescence frequency is calculated by the collision frequency and the coalescence efficiency which are based on the physical quantities, and most of them considered only the inertial collision caused by turbulent fluctuations (see Figure 6). Collision frequency models are classified into RC and WE, but both frequencies derive the mathematical functions with bubbles size, fluid properties (viscosity and turbulent energy dissipation rate) by experimental data or theoretical derivation in Figure 10. Collision efficiency models are divided into three models, namely, the energy model, film drainage model, as well as the critical velocity model.

(15) $$f_{c}=f_{coll}\eta_{c},$$

As previously stated, there are various mechanisms that promote the collision. Notwithstanding the fact that five mechanisms are listed [27], one main thing of the one-group interfacial area density transport equation is the turbulent random motion-induced collisions. The bubble collision is induced by the turbulent fluctuation around them. The concepts of collision cross-sectional area and bubble relative velocity $u_{\mathrm{rel}}$ are of leading importance. Kennard explained that collision efficiency is the effective volume swept by particles in unit time [45]. In order to determine the bubble relative velocity, Coulaloglou and Tavlarides [28], Lee et al. [32,33], Prince and Blanch [34], and Luo and Svendsen [36] regarded the colliding bubble velocity as the velocity of an equally sized eddy, in which way bubble relative velocity and collision frequency are obtained. It is necessary to apply three modification factors (see Table 3) into collision frequency. Although these modification factors are of help to obtain collision frequency, the necessity of Π and the formulation of Υ, Π still need for a further study, because the main difference in the models presents the results from the calculation of $u_{\mathrm{rel}}$ and modification factors Υ, Π. For example, Chesters [46] uses $u_{\mathrm{rel}}\propto {\left(D_{1}+D_{2}\right)}^{1/3}$ , and others use $u_{\mathrm{rel}}\propto {\left(D_{1}^{2/3}+D_{2}^{2/3}\right)}^{1/2}$ to get the value of $u_{\mathrm{rel}}$.

**Figure 11:**
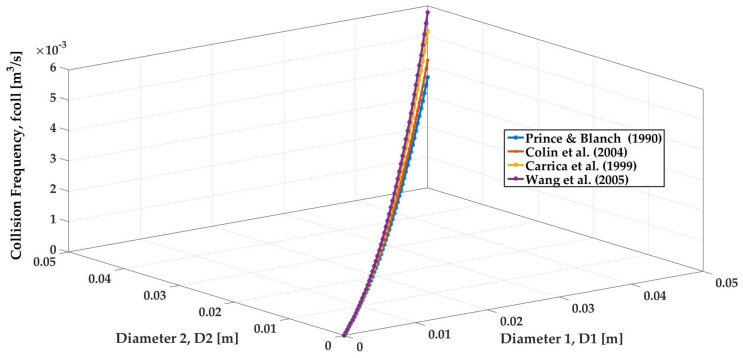
Trend of turbulent collision frequency on the bubble size, ε=1.

Turbulent collision frequencies of the dependent on the bubble sizes from the literature are shown in Figure 11. With the increase in bubble size ($D_{1}$ and $D_{2}$), the collision frequencies from all authors increase. Even though all authors take into account the buoyancy, shear rate, and wake interaction, the collision frequency of Wang et al.’s (the purple curve) [47,48] is the highest and twice the value of Prince and Blanch’s [34] (the blue one) due to an identical efficiency for all collision mechanisms by Prince and Blanch and the different forms for the different collision efficiency models by Wang et al. Moreover, Prince and Blanch do not take the cross-section of moving bubbles into consideration, while Wang et al. considers the modification factors Υ, Π and gives a small collision frequency for small bubbles due to the larger mean distance between small bubbles than big ones in the case of the same bubble number. Hence, Wang et al.’s model stacks up fully for their solid consideration of the modification factors as well as different collision frequencies of variable bubble size.

### 5.2. Constitutive Models for the One-Group IATE

One-group IATE formulations in Section 3.1 and its bubble coalescence and breakup frequency models in Section 5.1 have been proposed. Though a lot of efforts have been made to develop them in the past two decades, some obvious discrepancies exist in their constitutive models. Therefore, it is necessary to compare the typical expressions for bubble coalescence and breakup frequency and efficiency terms that have been commonly used in the literature. Analyzed on the constitutive models, frequency and efficiency of bubble coalescence and breakup obtained by these models are compared in this section.

The bubble coalescence and breakup constitutive models for the one-group IATE are usually developed from the experimental data taken in various flow conditions and flow channels. The salient findings of these studies are summarized in Table 4.

For the consideration in the modeling of bubble interaction mechanisms, Wu et al. set up bubble interaction mechanism terms, considering the contribution of RC, WE, and TI of the eddies against the bubbles [13]. In order to determine the adjustable parameters in the source and sink terms, experimental data of air-water upward flow in a 50.8 mm diameter pipe were used. Rather than WE, only RC and TI of the eddies against the bubbles were considered in the models of Hibiki and Ishii [49], and local flow measurements of vertical upward air-water flow in a round tube with an inner diameter of 50.8 mm were performed. Yao and Morel [18] took the contribution of RC and the TI of the eddies against the bubbles into consideration. Compared to previous studies, a significant modification made by Yao and Morel arises in two points: (a) bubble coalescence time = free traveling time + interaction time; (b) the breakup interaction time is modified by the bubble-eddy resonance mechanism. Furthermore, the contact time can be obtained from the characteristic time of eddies with the same radius of bubbles. Considering turbulent suppression phenomena in the turbulent bubbly flow with high velocity, Nguyen et al. [19] improved the RC model and the TI model proposed by Yao and Morel [18] and made a dynamical prediction of IAC evolution by the following RC model and TI model. The experimental conditions of Nguyen et al.’s work is the adiabatic air-water bubbly flow taken in vertical upward round pipes with inner diameters of 80 mm [50].

For the work on empirical coefficients, Ishii and Kim [16] developed a micro four-sensor conductivity probe and obtained the main parameters in radial distribution, providing the basis for studying the evolution of the two-phase flow. They enlarged the database for assessment of the source and sink terms proposed by Wu et al., simplified the evaluation procedure, and assumed that all parameters along the radial direction have uniform profiles, in which way the covariance of the terms under study can be neglected. Hence, a new set of empirical coefficients were presented by them. Applying the model by Wu et al. [13] into a 3D simulation of vertical upward two-phase flow in a pipeline using Fluent of CFD code, Wang [51] tested the primary model and proposed a set of new 3D simulation coefficients. The method for determining these coefficients is based on the experimental observation that different bubble interaction mechanisms dominate under different flow conditions. According to this thought, each coefficient can be estimated independently.

Although a lot of studies have been conducted on the IATE in the past two decades, further efforts should be made to improve the prediction accuracy and general applicability of the IATE. Some points can be summarized as follows:

1. Flow orientation and channel size:

As summarized in Table 4, nearly all the interfacial area transport data is derived from the vertical two-phase flows, hence the constitutive relations in vertical flow are fully-fledged compared to other flow directions. The latest applicability for horizontal flow was proposed by Talley [52]. Only one mechanism (TI) was observed in horizontal flow [52,53], while RC, TI, and WE were managed in vertical bubbly flow. It is necessary to model additional covariance parameters to consider the effects of non-uniform and asymmetric gas distribution in horizontal flow. Covariance parameters are coincident in vertical flow, but different for horizontal flow, owing to a skewed bubble distribution [54]. In order to calculate the covariance parameters, Talley proposed a void fraction profile reconstruction method to estimate the local void fraction [52], in which a 1D one-group IATE for horizontal flow was proved by the experiment with a small diameter horizontal pipe (38 mm) [52]. Yadav [55], Yadav et al. [56,57], Qiao [58], Qiao and Kim [59,60], and Qiao et al. [61] tried to study the interfacial structure in other orientation flows, but it was not enough. Although most of the work has focused on the straight pipe, two-phase flow undergoes various limitations such as elbows and tees in practice. Therefore, bubble coalescence and breakup IATE should be benchmarked by consistent data at various flow directions in a future study.

Kong [62,63] and Talley [52,53] thought that their work could be applied to different diameter pipes (38 mm, 51 mm, and 102 mm). As discussed above, the current interface area transport models are applied to a relatively small and limited channel with a diameter of 19.2~102 mm. With the decreasing of the channel size, RC may be restrained and WE may also become much more important. In short, channel size should be studied in a wide period.

2. Pressure and liquid type:

Water interfacial transport data derived from standard atmospheric pressure (0.1 MPa) is used for benchmarking existing sink and source terms, except for the boiling refrigerants and SF6-ethanol, where they can be used for the simulation of high pressure [64,65]. It is probably due to bubbles in water are common and used everywhere. As is known, bubbly flow with high pressure in industries, such as steam in the water of an electric power plant, oxygen gas in the hot metal of steelmaking, chlorine in sodium chloride molten salt system of titanium metallurgy, and carbon dioxide in methylamine of the chemical engineering, are widely used. Therefore, bubble studies under high pressure, metal liquid, and molten salt liquid should be carried out in the future for validation [66].

3. Simulation of developing and transient flow [65]:

Most of the interface area transport data is obtained at a certain distance from the inlet of the experiments section in steady flow as seen in Table 4. It means that the existing interface transport models have been verified under stable quasi-developing conditions, but not under transient flow conditions. The applicability of the interface transport model in developing and transient flow needs to be further studied.

**Figure 12:**
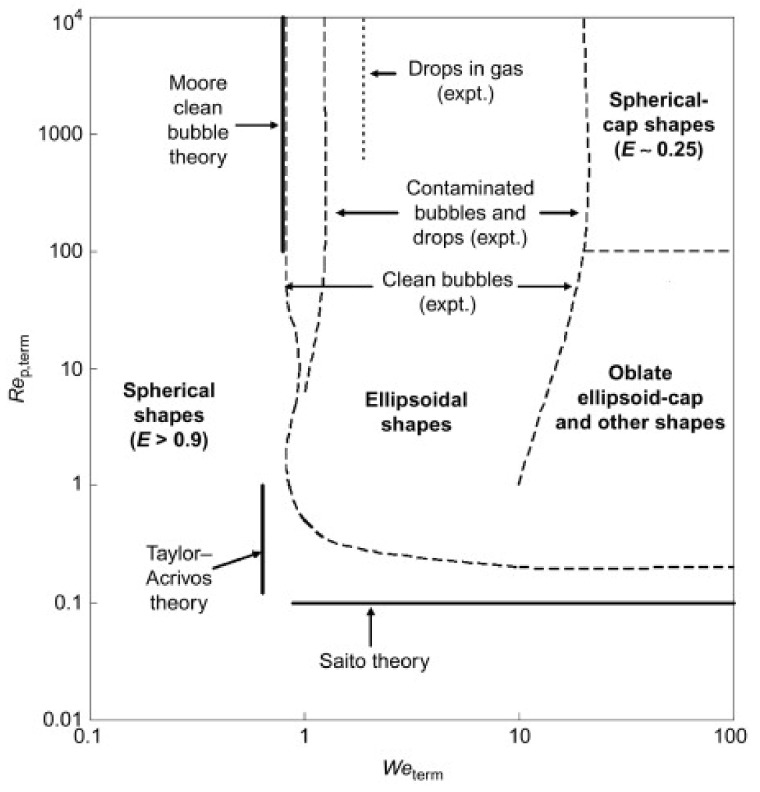
Expected bubble shapes, for different Reynolds and Weber numbers [55].

**Figure 13:**
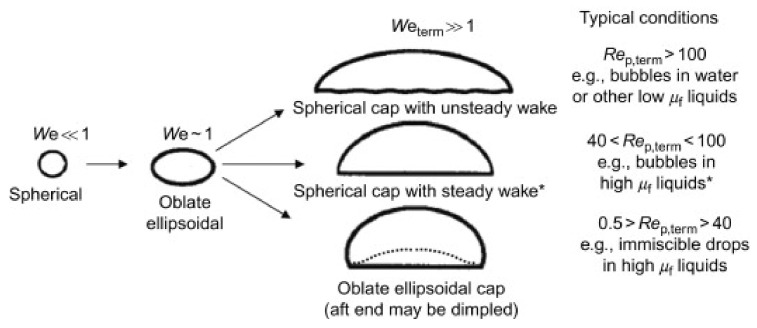
Examples of bubble shapes [55].

4. Bubble coalescence and breakup:

Compared with the dimensions in Table 4, bubble coalescence models were developed from 1D to 3D, from a simple constant (*C_RC_*) to a function of the Weber number. Furthermore, bubble breakup models were evaluated from the function of $\frac{We}{We_{cr}}$ and kept at a similar form for almost all the models. The steady-state shape of bubbles depends on the balance between drag and surface tension. For this reason, the Weber number has been used to map possible bubble shapes as shown in Figure 12 and Figure 13 [55].

Considering the coupling of the structure of air-water bubbly flow in a vertical pipe (volume fraction, energy dissipation rate, and bubble size distribution) [67], the evaluation of one-group constitutive models is presented in Section 5.2.1 and Section 5.2.2.

#### 5.2.1. Bubble Coalescence due to RC

**Figure 14:**
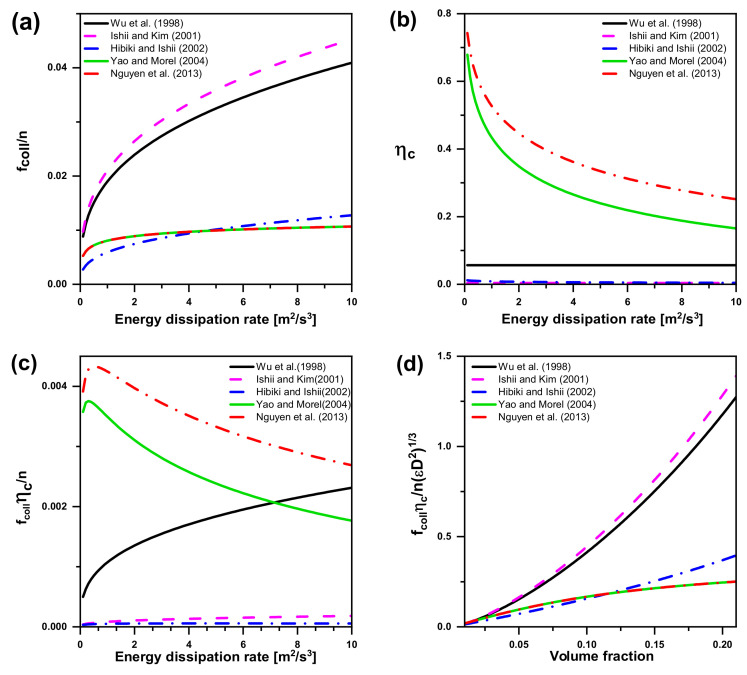
Bubble coalescence models in the literature. (**a**) Trend of the collision frequency as a function of the turbulent energy dissipation in bubble coalescence; (**b**) Trend of the coalescence efficiency as a function of the turbulent energy dissipation in bubble coalescence; (**c**) Trend of the collision frequency multiplied by the coalescence efficiency in bubble coalescence; (**d**) Trend of the collision models as a function of volume fraction in bubble coalescence.

As shown in Figure 14a, four random collision frequency models are compared. As summarized in Table 4, Nguyen et al.’s model is the same as Yao and Morel’s model, hence two curves (red and green) of the models are well coincident in $\frac{f_{coll}}{n}$. Although the modification factor functions vary which consider different maximum packing value, the expressions of the collision frequency reveal many differences vary from author to author. Yao and Morel employed a maximum packing value of 0.52, while previous investigators considered the value from 0.741 to 0.8. As Yao and Morel pointed out, the improvement of their model is due to the separate consideration of “the free travelling time” and “the interaction time” in the collision frequency term, thus, their model is slightly different from the former.

The trend of the coalescence efficiency as a function of the turbulent energy dissipation can be seen in Figure 14b, which varies significantly amongst different authors. In general, bubble coalescence occurs in the following process: (a) bubbles collide, (b) a little liquid between the bubbles is rejected and gradually drain, (c) when the film between bubbles reaches the critical thickness, the film breaks and bubble coalescence occurs. The results of the three models above are constant or decrease with the increases of turbulent energy dissipation. Significantly, Wu et al. did not apply a coalescence model, because the coalescence rate decreases exponentially with respect to the turbulent fluctuation velocity and different liquid flow conditions will lead to great differences. In order to describe the randomness of coalescence after each collision, a constant coefficient is used as the collision frequency $\eta_{c}$, which is not given explicitly but contained in $C_{RC}$. Regarding the coalescence efficiency as a constant, the models proposed by Wu et al. and Ishii and Kim are impractical. Nguyen et al. took turbulent suppression phenomena into consideration. They valued the adjustable coefficient $K_{c3}$ of 0.913, resulting in a little bit higher coalescence efficiency than the one proposed by Yao and Morel, which is regarded as a solid consideration.

The turbulent energy dissipation is a function of the random collision frequency multiplied by a constant coalescence efficiency. As can be seen in Figure 14c, the trend of the turbulent energy dissipation is affected by different proportionality coefficients chosen by the authors. Amongst the similar trend obtained by the models proposed by Wu et al., Ishii and Kim, and Hibiki and Ishii, the highest value is obtained by Wu et al.’s model and the slope of the curve is very high for very low values of the energy dissipation. Yet it is worth noting that there is a peak value with the turbulent energy dissipation of 0.3 and 0.5, respectively, both in the models proposed by Yao and Morel and Nguyen et al. and an opposite trend of the curves compared with Wu et al., Ishii and Kim, and Hibiki and Ishii.

As seen in Figure 14d, Ishii and Kim’s model obtained the highest value with the same trend as Wu et al.’s, as they chose coalescence efficiency as a constant and obtained similar collision frequency expressions. The intersection of Yao and Morel and Nguyen et al. is in the case of volume fraction valued 0.13 and both of them produce the upward trend lines with the low slopes. It is noteworthy that there is no significant relationship between the collision frequency and the critical Weber number.

#### 5.2.2. Bubble Breakup due to TI

**Figure 15:**
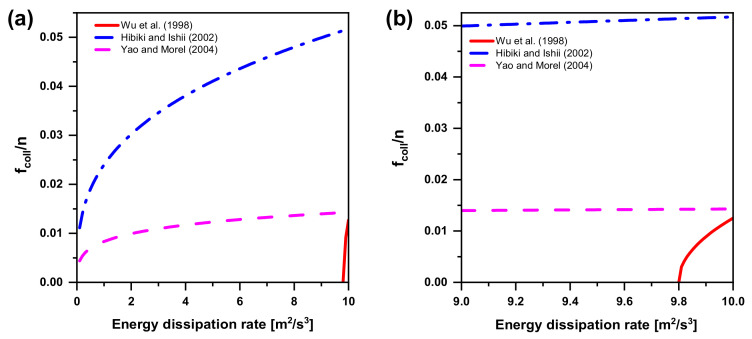
Bubble breakup models in the literature. (**a**) Trend of the collision frequency as a function of the turbulent energy dissipation in bubble breakup; (**b**) The scale range reduction in the collision frequency in bubble breakup.

Bubble breakup is considered to be due to the collision of turbulent eddies with bubbles. As for the effect of turbulent eddies on bubbles, all available bubble-eddy collision frequency and efficiency models can be compared, because the calculation formulation and almost all the coefficients have been given clearly. In Figure 15a, Wu et al.’s model does not present breakup events at all up to a critical value of the turbulent energy dissipation, where the critical value lies between 9 and 10 $m^{2}/s^{3}$. In order to realize the trend of models proposed by Wu et al., Hibiki and Ishii, and Yao and Morel, a scale range reduction is needed (see Figure 15b). In short, Wu et al.’s model cannot be applied to the flow with low turbulent dissipation (less than 9).

**Figure 16:**
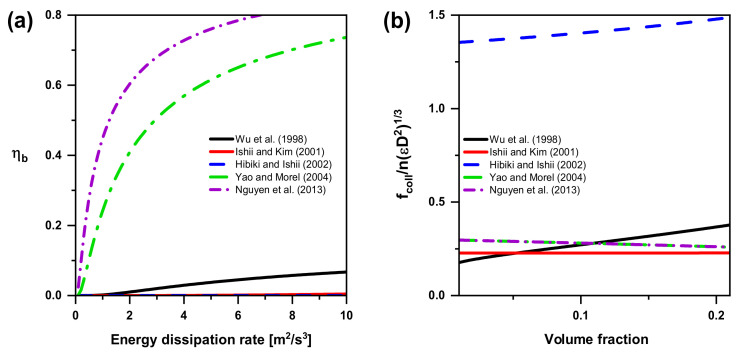
(**a**) Trend of the breakup efficiency as a function of the turbulent energy dissipation in bubble breakup; (**b**) Trend of the collision models as a function of volume fraction in bubble breakup.

As a function of turbulent energy dissipation, bubble breakup frequency models are compared in Figure 16a and the highest value is again obtained by Nguyen et al.’s model. It should be noted that there are the same trends in terms of bubble coalescence and breakup. The model delivering the second-highest rates is the one proposed by Yao and Morel. The curves of Ishii and Kim and Hibiki and Ishii’s models in Figure 16a are almost zero when turbulent energy dissipation is from zero to ten. It indicates turbulent energy dissipation has less effect on bubble breakup frequency (see $\eta_{b}$ in Table 4) and is opposite with the facts.

As seen in Figure 16b, Wu et al. and Ishii and Kim postulated that the bubble breakup will occur if We ≥ ${\mathrm{We}}_{cr}$. It means a turbulent eddy with enough energy to overcome the surface energy of interacting bubbles can break up a bubble. However, ${\mathrm{We}}_{cr}$ by Wu et al. is significantly different compared with the one by Ishii and Kim. Similar to what Yao and Morel proved, the bubble may distort owing to the interaction with a turbulent eddy with small energy, even if We < ${\mathrm{We}}_{cr}$. Moreover, Hibiki and Ishii proposed the infinite frequency in the case of α = $\alpha_{TI,\;max}$, where, in fact, the collision frequency should be zero for the reason that there is almost no liquid between bubbles and no turbulent eddy under this condition. With the improvement of Yao and Morel, their trend is close to the one by Ishii and Kim, the same as Nguyen et al., and an opposite trend with Wu et al.

However, the assumption-based values and the evaluation of model capability are not correlated. As a matter of fact, the source and sink terms explained in the previous sections are applied together, for each author, to modify the transported value in the IATE. Thus, the resultant value from the balance between creation and destruction of bubbles is what matters—the values of the single interaction terms are of secondary importance. Based on the discussion above, there are no perfect models for bubble coalescence and breakup. The best model for bubble coalescence due to RC is from Yao and Morel and Nguyen et al. ($\frac{f_{coll}}{n{\left(\varepsilon D^{2}\right)}^{1/3}}=\frac{\pi }{6}\left[\frac{K_{c1}\alpha \alpha_{max}^{1/3}}{\left(\alpha_{max}^{1/3}-\alpha^{1/3}\right)+K_{c2}\alpha_{max}^{1/3}\alpha \sqrt{\frac{We}{We_{cr}}}}\right]$ in Table 4) and the best one for bubble breakup due to TI is from Yao and Morel and Nguyen et al. ($\frac{f_{b,coll}}{n{\left(\varepsilon D^{2}\right)}^{1/3}}=\frac{\pi }{6}\left[\frac{C_{TI}\left(1-\alpha \right)}{1+K_{b2}\left(1-\alpha \right)\sqrt{\frac{We}{We_{cr}}}}\right]$ in Table 4), considering the sensibility of turbulent energy dissipation and efficiency in bubbly flow.

### 5.3. Constitutive Models for the Two-Group IATE

With the increase in bubble size, transport mechanisms become much more complicated because of the extra inner- and inter-group interaction mechanisms. Hence, two-group IATE for bubble coalescence and breakup is needed. In the gas-liquid flow beyond bubbly flow, the length scale of the Group II bubble can be comparable to the length scale of the flow channel. Therefore, two-group flow can be influenced by some geometry structures such as elbows and tees and the channel structure should be considered in the modeling and simulation. As the bubble size expands and reaches a limit point ($D_{cap,max}$ in Equation (6)), the gas-liquid interface will become unstable and no longer be sustained. Before the bubble size goes to $D_{cap,max}$, bubbles will break up due to SI. $D_{cap,max}$ proved by Miller et al. [68], is approximately 10 cm in an ambient air-water system.

Table 5 shows the existing constitutive models for two-group IATE in various conditions. All of them presented in the vertical upward air-water system study the modeling of five bubble interaction mechanisms (i.e., RC, WE, TI, SO, and SI) in two-group IATE.

In Table 5, in a narrow confined channel (10 mm × 200 mm), Sun [69] and Sun et al. [43] proposed a model of bubble interaction mechanisms in the two-group IATE for gas-liquid flow, including five major bubble interaction mechanisms (i.e., RC, WE, TI, SI, and SO). Bubbles are confined in the depth direction rather than the width direction due to the relatively high aspect ratio of the cross-section. There cannot be stable slug bubbles in such a channel, because the channel width is larger than $D_{cap,max}$ [43,69]. Later, they presented the evaluation approach and results of two-group IATE on experimental data obtained in confined upward flow [77]. The data derived from their results [77] and Ozar et al. [70] in an annular channel at elevated pressures (0.58 MPa) were evaluated for 1D two-group IATE. Yang et al. [71] developed Sun et al.’s model [43] and focused on the benchmark of IATE performance in a rod bundle geometry. The newly developed two-group model showed great prediction in bubbly flow to churn-turbulent flows. Results indicated that the average IAC and void fraction prediction error is less than 15% in most of the flow conditions [71].

In a round channel, pipe size affects the bubble interactions significantly. Fu [72] and Fu and Ishii [40] proposed a set of two-group IATE for moderate diameter round pipes (25 mm~100 mm). Solid databases for a two-inch air-water loop have been built [72] and experiments indicated that WE and SO are dominant in the bubble interaction mechanisms. Doup [78] and Worosz [23] improved Fu’s model and their predictions were in good agreement with experimental data. Wang et al. [24] established a database in a round pipe with a diameter of 25.4 mm ($D_{H}$ ≈ 2$D_{cap,max}$). Their model could not predict the drastic inter-group transfer and overestimated the IAC, hence extra experiments and modeling are necessary for the bubbly to slug transition flow in order to explain the sharp inter-group transfer in the two-phase flow. Furthermore, a more accurate model has been performed after the improvements including re-deriving the coalescence terms and optimizing the experimental coefficients.

In a relatively larger channel ($D_{H}$ > 2$D_{cap,max}$), Smith [73] developed a two-group IATE, and the model prediction was in good agreement with the experiments using a four-sensor conductivity probe in large diameter test sections, 101.6 mm and 152.4 mm diameter. Large bubbles will break up due to SI before they fill the pipe cross-section, hence, stable slug flow cannot exist in such a large pipe [79]. A modified model applicable to large pipes was developed by Smith [75] and Smith et al. [74], and two vertical test sections, one with a diameter of 102 mm and one with a diameter of 152 mm, were used in their work. In order to manage the poor predicting performance in interfacial area transport beyond the flow condition range in the original benchmarking effort, Schlegel et al. [76] modified the constitutive relations using an expanded range of experimental conditions extending to pipe sizes of 304 mm and the combined RMS error is below 15%.

In the past 20 years, substantial progress has been made in the simulation of bubbly, slug, and churn flow, but troubles in the experiment and simulation of churn-turbulent and annular flow. In the terms of modeling, the gas phase in churn-turbulent and annular flow is continuous, rather than discrete bubbles. Hence it is unreliable for investigators to set up models based on the previous work of bubble interaction mechanisms, and their experiments are difficult due to the highly distorted interface measurement. As discussed above, in order to expand the IATE to annular flow, the interface between the gas core and liquid film should be considered into IAC. Furthermore, the prediction ability of the 3D two-fluid model is a comprehensive coverage from bubbly to annular flow [54].

## 6. Conclusions and Outlook

Bubble interaction mechanisms play critical roles in both natural and industrial processes, of which constitutive models help to reveal, design, improve and optimize the bubble performance. To extend the IATE prediction capability beyond Group I bubbles in bubbly flow, two-group IATE which can deal with five bubble interaction mechanisms (i.e., RC, WE, TI, SO, and SI) is needed. However, to our knowledge, there is not yet a comprehensive review on both one-group and two-group IATE for bubble coalescence and breakup in the two-phase flow until now. Therefore, in the present work, bubble interaction mechanisms and their constitutive models of gas-liquid two-phase for both the one-group and two-group IATE are summarized and analyzed extensively from the literature. This includes transition phenomena from bubbly to turbulent-churn flows for bubble coalescence and breakup, IATE formulations, identification of bubble interaction mechanisms, bubble coalescence and breakup frequency models, and constitutive model development and evaluation. The following conclusions are highlighted.

1. Models for bubble coalescence and breakup processes of interfacial area density transport equation are based on the kinetic theory of gases. (1) The breakup frequency models are inconsistent and different models show completely different behaviors, where closure parameters change the conditions, such as integration limits, can have to be set. (2) In coalescence frequency models, the main difference in the models present results from the calculation of $u_{\mathrm{rel}}$ and modification factors Υ, Π which need further study to obtain collision frequency. Wang et al. gave a small collision frequency for small bubbles due to the larger mean distance between small bubbles than big ones in the case of the same bubble number [47,48].
2. Some important limitations of constitutive models for one-group IATE general applicability, such as flow orientation, channel size, pressure, liquid type, simulation of developing, and transient flow and bubble coalescence and breakup are summarized. Based on the summary and evaluation of the constitutive models for the one-group IATE in the literature, the best model for bubble coalescence due to RC is $\frac{f_{coll}}{n{\left(\varepsilon D^{2}\right)}^{1/3}}=\frac{\pi }{6}\left[\frac{K_{c1}\alpha \alpha_{max}^{1/3}}{\left(\alpha_{max}^{1/3}-\alpha^{1/3}\right)+K_{c2}\alpha_{max}^{1/3}\alpha \sqrt{\frac{We}{We_{cr}}}}\right]$, and the best one for bubble breakup due to TI is $\frac{f_{b,coll}}{n{\left(\varepsilon D^{2}\right)}^{1/3}}=\frac{\pi }{6}\left[\frac{C_{TI}\left(1-\alpha \right)}{1+K_{b2}\left(1-\alpha \right)\sqrt{\frac{We}{We_{cr}}}}\right]$ so far.
3. Constitutive models for two-group IATE in a three-type channel (i.e., narrow confined channel, round pipe, and relatively larger pipe) are reviewed, including five bubble interaction mechanisms, and their corresponding experimental conditions are summarized. Although great progress in extending the IATE beyond churn-turbulent flow to churn-annual flow was made, there is still some trouble in their modeling and experiments due to the highly distorted interface measurement.

For the future perspective of the IATE, its evolutions could be addressed in two different directions. On the one hand, it would be possible to complement the one-group IATE by means of the phase change and nucleation terms which makes the simulation of phase transition of subcooled boiling and saturated boiling regions possible, and the transition zone from bubbly to slug is avoided. On the other hand, as the adiabatic approximation is maintained, the two-group IATE should be considered and the modeling of other bubble interaction mechanisms is needed.

Significant efforts have been made to develop IATE over the past two decades, but this still requires further research to be conducted to improve the performance and make them applicable to a wider range of flow conditions. Therefore, inter- and inner-group terms should be proposed and the modification of the two-phase models is necessary. The inter-group transfers of mass momentum and eventually energy between group I and II bubbles need to be taken into account. Furthermore, the modeling of bubble interactions for two-group IATE is strongly dependent on the channel size and geometry. Hence different flow restrictions (elbows, tees, etc.) and flow orientations (horizontal and inclined) need to be studied in the future for the application of real life, where extensive measurements and experimental data are essential for the validation under different flow conditions. The smooth transition from one-group to two-group IATE, that is, from bubbly to turbulent-churn flow, needs to be further improved.

## Figures and Tables

**Table 1:** Breakup criterion of several literature [27].

Criterion	Authors
(a) Turbulent kinetic energy of the particle greater than a critical value	Coulaloglou and Tavlaride [28]
	Chatzi et al. [29]
(b) Velocity fluctuation around the particle surface greater than a critical value	Narsimhan and Ghupta [30]
	Alopaeus et al. [31]
(c) Turbulent kinetic energy of the hitting eddy greater than a critical value	Lee et al. [32,33]
	Prince and Blanch [34]
	Tsouris and Tavlarides [35]
	Luo and Svendsen [36]
	Martinez Bazan et al. [37]
(d) Inertial force of the hitting eddy greater than the interfacial force of the smallest daughter particle	Lehr and Mewes [38]
	Lehr et al. [39]
(e) Combination of (c) and (d)	Wang et al. [25]
	Zhao and Ge [26]

**Table 2:** Major bubble interaction mechanisms [43].

Mechanism	Interaction	$\alpha_{i}\mathrm{Source}/\mathrm{Sink}\;\mathrm{Term}$	$\alpha_{i}\mathrm{Source}/\mathrm{Sink}$
RC	(1)+(1)→(1)	$\Phi_{RC}^{\left(1\right)}$	Sink in 1
	(1)+(1)→(2)	$\Phi_{RC}^{\left(11,2\right)}$	Sink in 1; source in 2
	(1)+(2)→(2)	$\Phi_{RC}^{\left(12,2\right)}$	Sink in 1; source in 2 (no number change)
	(2)+(2)→(2)	$\Phi_{RC}^{\left(2\right)}$	Sink in 2
WE	(1)+(1)→(1)	$\Phi_{WE}^{\left(1\right)}$	Sink in 1
	(1)+(1)→(2)	$\Phi_{WE}^{\left(11,2\right)}$	Sink in 1; source in 2
	(1)+(2)→(2)	$\Phi_{WE}^{\left(12,2\right)}$	Sink in 1; source in 2 (no number change)
	(2)+(2)→(2)	$\Phi_{WE}^{\left(2\right)}$	Sink in 2
TI	(1)→(1)+(1)	$\Phi_{TI}^{\left(1\right)}$	Source in 1
	(2)→(1)+(1)	$\Phi_{TI}^{\left(2,11\right)}$	Source in 1; sink in 2
	(2)→(2)+(1)	$\Phi_{TI}^{\left(2,12\right)}$	Source in 1; sink in 2 (no number change)
	(2)→(2)+(2)	$\Phi_{TI}^{\left(2\right)}$	Source in 2
SO	(2)→(2)+n(1)	$\Phi_{SO}^{\left(2,12\right)}$	Source in 1 (multiple number); sink in 2 (no number change)
SI	(2)→(2)+(2)	$\Phi_{SI}^{\left(2\right)}$	Source in 2

**Table 3:** Modification factors in coalescence models.

Modification Factor	Definition
Λ	The effect of the size ratio between bubbles and eddies.
Υ	The effect of the bubble that reduces the free space for bubble movement and causes an increase in the collision frequency.
Π	The ratio of the mean distance between bubbles to their average relative turbulent path length.

**Table 4:** Existing constitutive models for one-group IATE.

Investigators	Flow Orientation	Dimensions	Channel Size (mm)	Pressure (MPa)	System	Bubble Coalescence		Bubble Breakup	
						Collision Frequency	Efficiency	Collision Frequency	Efficiency
Wu et al. (1998) [13]	Upward	1D	50.8	0.1	Air-water	$\frac{f_{coll}}{n{\left(\varepsilon D^{2}\right)}^{1/3}}=\frac{6\alpha }{\pi \alpha_{max}^{1/3}\left(\alpha_{max}^{1/3}-\alpha^{1/3}\right)}\left[1-\exp\left(-C\frac{\alpha_{max}^{1/3}\alpha^{1/3}}{\alpha_{max}^{1/3}-\alpha^{1/3}}\right)\right]$ $C=3.0,\;\alpha_{max}=0.8$	$\eta_{c}=C_{RC}=0.0565$	$\frac{f_{b,coll}}{n{\left(\varepsilon D^{2}\right)}^{1/3}}=\frac{1}{\alpha_{max}^{1/3}\left(\alpha_{max}^{1/3}-\alpha^{1/3}\right)}{\left(1-\frac{We_{cr}}{We}\right)}^{1/2}$ $We_{cr}=2.0,$ $\;\alpha_{max}=0.8$	$\eta_{b}=C_{TI}exp\left(-\frac{We_{cr}}{We}\right)$ $We_{cr}=2.0,\;C_{TI}=0.18$
Ishii and Kim (2001) [16]	Upward	1D	25.4/50.8/101.6	0.1	Air-water	$\frac{f_{coll}}{n{\left(\varepsilon D^{2}\right)}^{1/3}}=\frac{6\alpha }{\pi \alpha_{max}^{1/3}\left(\alpha_{max}^{1/3}-\alpha^{1/3}\right)}\left[1-\exp\left(-C\frac{\alpha_{max}^{1/3}\alpha^{1/3}}{\alpha_{max}^{1/3}-\alpha^{1/3}}\right)\right]$ $C=3.0,\;\alpha_{max}=0.75$	$\eta_{c}=C_{RC}=0.004$	$\frac{f_{b,coll}}{n{\left(\varepsilon D^{2}\right)}^{1/3}}=\frac{1}{\alpha_{max}^{1/3}\left(\alpha_{max}^{1/3}-\alpha^{1/3}\right)}{\left(1-\frac{We_{cr}}{We}\right)}^{1/2}$ $We_{cr}=6.0,$ $\alpha_{max}=0.75$	$\eta_{b}=C_{TI}exp\left(-\frac{We_{cr}}{We}\right)$ $We_{cr}=6.0,\;C_{TI}=0.085$
Hibiki and Ishii (2002) [17]	Upward	1D	25.4/50.8	0.1	Air-water	$\frac{f_{coll}}{n{\left(\varepsilon D^{2}\right)}^{1/3}}=\frac{\alpha }{\left(\alpha_{RC,\;max}-\alpha \right)}$ $\alpha_{RC,\;max}=0.741$	$\eta_{c}=\frac{C_{RC}}{2}exp\left(-K_{c}\sqrt{\frac{We}{2}}\right)$ $C_{RC}=0.0314,\;K_{c}=1.29$	$\frac{f_{b,coll}}{n{\left(\varepsilon D^{2}\right)}^{1/3}}=\frac{1-\alpha }{\left(\alpha_{TI,\;max}-\alpha \right)}$ $\alpha_{TI,\;max}=0.741$	$\eta_{b}=C_{TI}exp(-\frac{2K_{TI}}{We}$) $K_{TI}=6.0,\;C_{TI}=0.021$
Yao and Morel (2004) [18]	Upward	3D	19.2	1.46–26.17	Air-dichlorodifluoromethane	$\frac{f_{coll}}{n{\left(\varepsilon D^{2}\right)}^{1/3}}=\frac{\pi }{6}\left[\frac{K_{c1}\alpha \alpha_{max}^{1/3}}{\left(\alpha_{max}^{1/3}-\alpha^{1/3}\right)+K_{c2}\alpha_{max}^{1/3}\alpha \sqrt{\frac{We}{We_{cr}}}}\right]$ $K_{c1}=2.86,\;K_{c2}=1.922,\;We_{cr}=1.24,\;\alpha_{max}=0.52$	$\eta_{c}=exp\left(-K_{c3}\sqrt{\frac{We}{We_{cr}}}\right)$ $K_{c3}=1.017,\;We_{cr}=1.24$	$\frac{f_{b,coll}}{n{\left(\varepsilon D^{2}\right)}^{1/3}}=\frac{\pi }{6}\left[\frac{C_{TI}\left(1-\alpha \right)}{1+K_{b2}\left(1-\alpha \right)\sqrt{\frac{We}{We_{cr}}}}\right]$ $C_{TI}=1.6,\;K_{b2}=0.42,\;We_{cr}=1.24$	$\eta_{b}=exp\left(-\frac{We_{cr}}{We}\right)$ $We_{cr}=1.24$
Nguyen et al. (2013) [19]	Upward	3D	80	0.2	Air-water	$\frac{f_{coll}}{n{\left(\varepsilon D^{2}\right)}^{1/3}}=\frac{\pi }{6}\left[\frac{K_{c1}\alpha \alpha_{max}^{1/3}}{\left(\alpha_{max}^{1/3}-\alpha^{1/3}\right)+K_{c2}\alpha_{max}^{1/3}\alpha \sqrt{\frac{We}{We_{cr}}}}\right]$ $K_{c1}=2.86,\;K_{c2}=1.922,\;We_{cr}=1.24,\;\alpha_{max}=0.52$	$\eta_{c}=exp\left(-\frac{K_{c3}}{k^{2/3}}\sqrt{\frac{We}{2}}\right)$ $K_{c3}=0.913,\;We_{cr}=1.24$	$\frac{f_{b,coll}}{n{\left(\varepsilon D^{2}\right)}^{1/3}}=\frac{\pi }{6}\left[\frac{C_{TI}\left(1-\alpha \right)}{1+K_{b2}\left(1-\alpha \right)\sqrt{\frac{We}{We_{cr}}}}\right]$ $C_{TI}=1.6,\;K_{b2}=0.42,\;We_{cr}=1.24$	$\eta_{b}=exp\left(-\frac{K_{b3}}{1-k_{1}}\frac{2}{We}\right)$ $K_{b3}=1.59,\;We_{cr}=1.24$

**Table 5:** Existing constitutive models for two-group IATE in various conditions.

Channel Type	Investigators	Channel Size [mm]	Flow Conditions	Bubble Coalescence		Bubble Breakup		
				RC	WE	TI	SO	SI/Pressure Change
Narrow confined channel	Sun et al. (2004) [43,69]	10 × 200	0.1 MPa	$\Phi_{RC}^{\left(1\right)}=-0.17C_{RC}^{\left(1\right)}\eta_{RC}^{\left(1\right)}\frac{\varepsilon^{\frac{1}{3}}\alpha_{1}a_{i1}^{\frac{5}{3}}}{\alpha_{1,max}^{\frac{1}{3}}\left(\alpha_{1,max}^{\frac{1}{3}}-\alpha_{1}^{\frac{1}{3}}\right)}\times \left[1-exp\left(-C_{RC1}\frac{\alpha_{1,max}^{\frac{1}{3}}\alpha_{1}^{\frac{1}{3}}}{\alpha_{1,max}^{\frac{1}{3}}-\alpha_{1}^{\frac{1}{3}}}\right)\right]$ $\Phi_{RC}^{\left(11,2\right)}=0.68C_{RC}^{\left(1\right)}\frac{\varepsilon^{\frac{1}{3}}\alpha_{1}^{2}a_{i1}^{\frac{2}{3}}}{\alpha_{1,max}^{\frac{2}{3}}G}\times \left[1-exp\left(-C_{RC1}\frac{\alpha_{1,max}^{\frac{1}{3}}\alpha_{1}^{\frac{1}{3}}}{\alpha_{1,max}^{\frac{1}{3}}-\alpha_{1}^{\frac{1}{3}}}\right)\right]\left[1+0.7G^{7/6}{\left(\frac{a_{i1}}{\alpha_{1}}\right)}^{1/2}{\left(\frac{\sigma }{g\Delta \rho }\right)}^{-1/3}\right]\left(1-\frac{2}{3}D_{c1}^{*}\right)$ $\Phi_{RC,1}^{\left(12,2\right)}=-4.85C_{RC}^{\left(12,2\right)}\frac{\varepsilon^{\frac{1}{3}}\alpha_{1}^{\frac{2}{3}}\alpha_{2}^{2}a_{i1}}{R_{t,max2}^{\frac{2}{3}}}\times \left[1-exp\left(-C_{RC1}\frac{\alpha_{1,max}^{\frac{1}{3}}\alpha_{1}^{\frac{1}{3}}}{\alpha_{1,max}^{\frac{1}{3}}-\alpha_{1}^{\frac{1}{3}}}\right)\right]$ $\Phi_{RC,2}^{\left(12,2\right)}=13.6C_{RC}^{\left(12,2\right)}\frac{\varepsilon^{\frac{1}{3}}\alpha_{1}^{\frac{5}{3}}\alpha_{2}^{2}}{R_{t,max2}^{\frac{2}{3}}G}\left(1+\frac{10.3G}{R_{t,max2}^{\frac{2}{3}}}\right)\times \left[1-exp\left(-C_{RC1}\frac{\alpha_{1,max}^{\frac{1}{3}}\alpha_{1}^{\frac{1}{3}}}{\alpha_{1,max}^{\frac{1}{3}}-\alpha_{1}^{\frac{1}{3}}}\right)\right]$ $\Phi_{RC}^{\left(2\right)}=-13.6C_{RC}^{\left(2\right)}\frac{\varepsilon^{\frac{1}{3}}\alpha_{2}^{2}R_{t,max2}^{\frac{4}{3}}}{W^{2}G}\times \left[1-exp\left(-C_{RC2}\alpha_{2}^{\frac{1}{2}}\right)\right]\;\left(1-2.0R_{t,c2}^{*}{}^{2}+\frac{9.0G}{R_{t,max2}}\right)$	$\Phi_{WE}^{\left(1\right)}=-0.27C_{WE}^{\left(1\right)}C_{D1}^{1/3}v_{r1}a_{i1}^{2}$ $\Phi_{WE,2}^{\left(11,2\right)}=1.08C_{WE}^{\left(11,2\right)}C_{D1}^{\frac{1}{3}}v_{r1}a_{i1}^{2}\left(1-\frac{2}{3}D_{c1}^{*}\right)\frac{\alpha_{1}a_{i1}}{G}\left[1+0.7G^{\frac{7}{6}}{\left(\frac{a_{i1}}{\alpha_{1}}\right)}^{\frac{1}{2}}{\left(\frac{\sigma }{g\Delta \rho }\right)}^{-\frac{1}{3}}\right]$ $\Phi_{WE,1}^{\left(12,2\right)}=-4.35C_{WE}^{\left(12,2\right)}\sqrt{gC_{D2}G}\frac{\alpha_{2}a_{i1}}{R_{t,max2}}$ $\Phi_{WE,2}^{\left(12,2\right)}=26.1C_{WE}^{\left(12,2\right)}a_{1}a_{2}\sqrt{\frac{gC_{D2}}{G}}\frac{1}{R_{t,max2}}\left(1+4.31\frac{G}{R_{t,max2}}\right)$ $\Phi_{WE}^{\left(2\right)}=-15.9C_{WE}^{\left(2\right)}\frac{\alpha_{2}^{2}}{R_{t,max2}^{2}}\left(1+0.51R_{t,cr}^{*}\right)$	$\Phi_{TI}^{\left(1\right)}=0.12C_{TI}^{\left(1\right)}\varepsilon^{\frac{1}{3}}\left(\frac{a_{i1}^{\frac{5}{3}}}{\alpha_{1}^{\frac{2}{3}}}\right)\left(1-\alpha \right)exp\left(-\frac{We_{cr1}}{We_{1}}\right)\sqrt{1-\frac{We_{cr1}}{We_{1}}}$ $\Phi_{TI,1}^{\left(2,1\right)}=2.71C_{TI}^{\left(2\right)}\frac{\varepsilon^{\frac{1}{3}}G^{\frac{2}{3}}R_{t}^{*\frac{5}{3}}\left(1-R_{t,cr}^{*\frac{5}{3}}\right)}{R_{t,max2}^{7/3}}\alpha_{2}\left(1-\alpha \right)exp\left(-\frac{We_{cr2}}{We_{2}}\right)\sqrt{1-\frac{We_{cr2}}{We_{2}}}$ $\Phi_{TI,2}^{\left(2\right)}=1.4C_{TI}^{\left(2\right)}\frac{\varepsilon^{\frac{1}{3}}G\left(1-2R_{t,cr}^{*}\right)}{R_{t,max2}^{8/3}}\alpha_{2}\left(1-\alpha \right)exp\left(-\frac{We_{cr2}}{We_{2}}\right)\sqrt{1-\frac{We_{cr2}}{We_{2}}}$	$\Phi_{SO,1}^{\left(2,12\right)}=64.51C_{SO}C_{D}^{2}\frac{\alpha_{2}v_{rb}}{GR_{t,max2}}\left[1-{\left(\frac{We_{c,SO}}{We_{max2}}\right)}^{3}\right]$ $\Phi_{SO,2}^{\left(2,12\right)}=-21.50C_{SO}C_{D}^{3}{\left(\frac{\sigma }{\rho_{f}}\right)}^{\frac{3}{5}}\frac{\alpha_{2}}{G^{\frac{8}{5}}R_{t,max2}v_{rb}^{\frac{1}{5}}}\left[1-{\left(\frac{We_{c,SO}}{We_{max2}}\right)}^{3}+\frac{3.24G}{R_{t,max2}}\left(1-{\left(\frac{We_{c,SO}}{We_{max2}}\right)}^{2}\right)\right]$	$\Phi_{SI}^{\left(2\right)}\approx 1.25\alpha_{2}^{2}{\left(\frac{\sigma }{g\Delta \rho }\right)}^{-1}\left\{C_{RC}^{\left(2\right)}\frac{\varepsilon^{\frac{1}{3}}}{W^{2}}{\left(\frac{\sigma }{g\Delta \rho }\right)}^{\frac{7}{6}}\left[1-\exp\left(-C_{RC2}\alpha_{2}^{\frac{1}{2}}\right)\right]+2.3\times 10^{-4}C_{WE}^{\left(2\right)}\sqrt{gC_{D}G}\right\}$
	Ozar et al. (2013) [70]	Annular: 19.1 (inner)/38 (outer)	0.58 MPa,$j_{f}$ = 0.23~3.31 m/s, $j_{g}$ = 0.04~3.06 m/s					
	Yang et al. (2016) [71]	10.3	0.1~0.3 MPa					
Round channel	Fu (2001) [72], Fu & Ishii (2003) [40]	25~100	0.1 MPa	$\Phi_{RC}^{\left(1\right)}=R_{RC}^{\left(1\right)}D_{sm1}^{2}\left[-3.142D_{c1}^{*}{}^{3}+2.183D_{c1}^{*}{}^{5}-0.395D_{c1}^{*}{}^{8}+3.392\left(0.579D_{c1}^{*}{}^{3}-1\right)\right]$ $\Phi_{RC,1}^{\left(11,2\right)}=R_{RC}^{\left(1\right)}D_{sm1}^{2}\left[8.82+2.035{\left(0.579D_{c1}^{*}{}^{3}-1\right)}^{\frac{8}{3}}-5.428D_{c1}^{*}{}^{3}\right]$ $\Phi_{RC,2}^{\left(11,2\right)}=R_{RC}^{\left(1\right)}D_{sm1}^{2}\left(6.462-2.182D_{c1}^{*}{}^{5}+0.395D_{c1}^{*}{}^{8}\right]$ where $R_{RC}^{\left(1\right)}=C_{RC}^{\left(1\right)}\frac{In_{1}^{2}D_{sm1}^{2}}{\alpha_{1,max}^{\frac{1}{3}}\left(\alpha_{1,max}^{\frac{1}{3}}-\alpha_{1}^{\frac{1}{3}}\right)}\times [1-exp\left(-C_{RC1}\frac{\alpha_{1,max}^{\frac{1}{3}}\alpha_{1}^{\frac{1}{3}}}{\alpha_{1,max}^{\frac{1}{3}}(\alpha_{1,max}^{\frac{1}{3}}-\alpha_{1}^{\frac{1}{3}})}\right)$	$\Phi_{WE}^{\left(1\right)}=R_{WE}^{\left(1\right)}D_{sm1}^{2}\left[-3.142D_{c1}^{*}{}^{3}+2.183D_{c1}^{*}{}^{5}-0.395D_{c1}^{*}{}^{8}+3.392\left(0.579D_{c1}^{*}{}^{3}-1\right)\right]$ $\Phi_{WE,1}^{\left(11,2\right)}=R_{WE}^{\left(1\right)}D_{sm1}^{2}\left[8.82+2.035{\left(0.579D_{c1}^{*}{}^{3}-1\right)}^{\frac{8}{3}}-5.428D_{c1}^{*}{}^{3}\right]$ $\Phi_{WE,2}^{\left(11,2\right)}=2{\left(1-0.2894D_{c1}^{*}{}^{3}\right)}^{2}\times R_{WE}^{\left(1\right)}D_{sm1}^{2}\left(6.462-2.182D_{c1}^{*}{}^{5}+0.395D_{c1}^{*}{}^{8}\right]$ $\Phi_{WE}^{\left(2\right)}=-10.24C_{WE}^{\left(2\right)}D^{\frac{3}{2}}\alpha_{2}\left[1-\exp\left(\frac{-2331\alpha_{2}V_{s}^{*2}}{D^{5}}\right)\right]{\left[\exp\left(\frac{-0.06C_{1}\left(\frac{\alpha_{2m}}{\alpha_{2}}-1\right)}{V_{s}^{*}}\right)-1\right]}^{-1}$ $\Phi_{WE,1}^{\left(12,2\right)}=-3\pi C_{WE}^{\left(12,2\right)}D^{1/2}{\left(\frac{2g\Delta \rho }{\rho_{f}}\right)}^{1/2}V_{s}^{*1/2}\frac{\alpha_{1}\alpha_{2}}{1-\alpha_{2}}\kappa_{fr}D_{sm1}^{-1}$ $\Phi_{WE,2}^{\left(12,2\right)}=2\pi C_{WE}^{\left(12,2\right)}D^{-1/2}\alpha_{2m}^{-1/2}{\left(\frac{2g\Delta \rho }{\rho_{f}}\right)}^{1/2}V_{s}^{*1/2}\frac{\alpha_{1}\alpha_{2}}{1-\alpha_{2}}\kappa_{fr}$ where $R_{WE}^{\left(1\right)}=C_{WE}^{\left(1\right)}C_{d}^{\frac{1}{3}}n_{1}^{2}D_{sm1}^{2}v_{r1}$, $v_{r1}=\frac{gD_{sm1}\Delta \rho }{3C_{d}\rho_{f}}$, $C_{d}=\frac{2}{3}D_{sm1}\sqrt{\frac{g\Delta \rho }{\sigma }}{\left[\frac{1+17.67{\left(1-\alpha_{1}\right)}^{2.6}}{18.67{\left(1-\alpha_{1}\right)}^{3}}\right]}^{3}$	$\Phi_{TI}^{\left(1\right)}=\frac{1}{18}C_{TI}^{\left(1\right)}\left(\frac{Ia_{i1}^{2}}{\alpha_{1}}\right)exp\left(-\frac{We_{cr}}{We^{*}}\right)\sqrt{1-\frac{We_{cr}}{We^{*}}}$ for $We^{*}>We_{cr1}$, otherwise $\Phi_{TI}^{\left(1\right)}=0$.	$\Phi_{SO,1}^{\left(2,1\right)}=0.5755C_{SO}v_{g}^{0.5}C_{g}^{2}{\left(\frac{\rho_{f}}{\sigma D}\right)}^{\frac{3}{5}}\alpha_{2}V_{s}^{*-\frac{4}{5}}\left(1-0.6535\kappa_{bl}\right)\xi_{SO}\kappa_{fr}^{2}$ $\Phi_{SO,2}^{\left(2,1\right)}=-4.4332C_{SO}v_{g}^{0.5}D^{-9/51/2}C_{g}^{4/5}\alpha_{2m}^{1/2}\alpha_{2}V_{s}^{*-\frac{1}{5}}\left(1-0.6474\kappa_{bl}\right)\kappa_{fr}^{4/5}$ where $\xi_{SO}={\left[1-\exp\left(-\gamma_{SO}\left(\frac{\alpha_{2,max}}{\alpha_{2,max}-\alpha_{2}}\right)\beta_{SO}\frac{We_{cr}}{We_{1}}\right)\right]}^{-1}$	$\Phi_{exp,1}=-\frac{2}{3}\frac{a_{i1}v_{g1}}{p}\frac{\partial p}{\partial z}$ $\Phi_{exp,2}=-\left(\frac{4+10.19V_{s}^{*2}}{\alpha_{2m}^{0.5}D}\right)\frac{a_{i2}v_{g2}}{p}\frac{\partial p}{\partial z}$ $\Phi_{exp,12}=\zeta D_{c1}^{*}{}^{2}\frac{a_{i1}}{\alpha_{1}}\frac{1}{1-CD_{c1}^{*}{}^{3}}\left(\nabla \cdot \left(\alpha_{1}v_{g1}\right)-\sum_{j}\eta_{j,1}^{inter}\right)$ where $\zeta =0.00444{\left(\frac{D_{sm1}}{D_{cap,max}}\right)}^{0.36}\alpha_{1}{}^{-1.35}$, $\sum_{j}\eta_{j,1}^{inter}=-\left(\eta_{RC}^{\left(11,2\right)}+\eta_{WE}^{\left(11,2\right)}+\eta_{WE}^{\left(12,2\right)}+\eta_{SO}^{\left(2,1\right)}\right)$
	Woroz (2015) [23]	50.8	0.1 MPa					
	Wang et al. (2019) [24]	25.4	0.1 MPa					
Relatively larger channel ($D_{H}$>2$D_{cap,max}$)	Smith (2002) [73]	101.6/152.4	0.1 MPa	$\Phi_{RC}^{\left(1\right)}=-0.17C_{RC}^{\left(1\right)}\eta_{RC}^{\left(1\right)}\frac{\varepsilon^{\frac{1}{3}}\alpha_{1}a_{i1}^{\frac{5}{3}}}{\alpha_{1,max}^{\frac{1}{3}}\left(\alpha_{1,max}^{\frac{1}{3}}-\alpha_{1}^{\frac{1}{3}}\right)}\times \left[1-exp\left(-C_{RC1}\frac{\alpha_{1,max}^{\frac{1}{3}}\alpha_{1}^{\frac{1}{3}}}{\alpha_{1,max}^{\frac{1}{3}}-\alpha_{1}^{\frac{1}{3}}}\right)\right]$ $\Phi_{RC}^{\left(11,2\right)}=4.1C_{RC}^{\left(1\right)}\eta_{RC}^{\left(1\right)}\frac{\varepsilon^{\frac{1}{3}}\alpha_{1}a_{i1}^{\frac{5}{3}}}{\alpha_{1,max}^{\frac{2}{3}}}\times \left[1-exp\left(-C_{RC1}\frac{\alpha_{1,max}^{\frac{1}{3}}\alpha_{1}^{\frac{1}{3}}}{\alpha_{1,max}^{\frac{1}{3}}-\alpha_{1}^{\frac{1}{3}}}\right)\right]\left(1-\frac{2}{3}D_{c1}^{*}\right)$ $\Phi_{RC,1}^{\left(12,2\right)}=-1.14C_{RC}^{\left(12,2\right)}\eta_{RC}^{\left(12,2\right)}\varepsilon^{\frac{1}{3}}\alpha_{1}^{\frac{2}{3}}\alpha_{2}^{\frac{4}{3}}a_{i1}a_{i2}^{\frac{2}{3}}\times \left[1-exp\left(-C_{RC1}\frac{\alpha_{1,max}^{\frac{1}{3}}\alpha_{1}^{\frac{1}{3}}}{\alpha_{1,max}^{\frac{1}{3}}-\alpha_{1}^{\frac{1}{3}}}\right)\right]$ $\Phi_{RC,2}^{\left(12,2\right)}=1.80C_{RC}^{\left(12,2\right)}\eta_{RC}^{\left(12,2\right)}\varepsilon^{\frac{1}{3}}\alpha_{1}^{\frac{5}{3}}\alpha_{2}^{\frac{1}{3}}\alpha_{i1}a_{i2}^{\frac{5}{3}}\times \left[1-exp\left(-C_{RC1}\frac{\alpha_{1,max}^{\frac{1}{3}}\alpha_{1}^{\frac{1}{3}}}{\alpha_{1,max}^{\frac{1}{3}}-\alpha_{1}^{\frac{1}{3}}}\right)\right]$ $\Phi_{RC}^{\left(2\right)}=-95.7C_{RC}^{\left(2\right)}\eta_{RC}^{\left(2\right)}\frac{\varepsilon^{\frac{1}{3}}\alpha_{2}^{\frac{7}{3}}}{D_{H}^{2}a_{i2}^{\frac{1}{3}}}\times \left[1-exp\left(-C_{RC2}\alpha_{2}^{\frac{1}{2}}\right)\right]\;\left(1-0.37D_{c2}^{*}{}^{3}\right)$	$\Phi_{WE}^{\left(1\right)}=-0.17C_{WE}^{\left(1\right)}C_{D1}^{1/3}v_{r1}a_{i1}^{2}$ $\Phi_{WE,2}^{\left(11,2\right)}=2.57C_{WE}^{\left(11,2\right)}C_{D1}^{1/3}v_{r1}a_{i1}^{2}\left(1-\frac{2}{3}D_{c1}^{*}\right)$ $\Phi_{WE,l1}^{\left(12,2\right)}=-0.33C_{WE}^{\left(12,2\right)}{\bar{u}}_{w12}a_{i1}a_{i2}$ $\Phi_{WE,g1}^{\left(12,2\right)}=0.922C_{WE}^{\left(12,2\right)}{\bar{u}}_{w12}\alpha_{1}\frac{a_{i2}^{2}}{\alpha_{2}}$ $\Phi_{WE}^{\left(2\right)}=-0.102C_{WE}^{\left(2\right)}\left[1-\exp\left(-0.7\alpha_{2}\right)\right]{\bar{u}}_{rw2}\frac{a_{i2}^{2}}{\alpha_{2}}\left(1-0.10D_{c2}^{*}{}^{2}\right)$	$\Phi_{TI}^{\left(1\right)}=0.12C_{TI}^{\left(1\right)}\varepsilon^{\frac{1}{3}}\left(\frac{a_{i1}^{\frac{5}{3}}}{\alpha_{1}^{\frac{2}{3}}}\right)\left(1-\alpha \right)exp\left(-\frac{We_{cr1}}{We_{1}}\right)\sqrt{1-\frac{We_{cr1}}{We_{1}}}$ $\Phi_{TI,g1}^{\left(2,1\right)}=6.165C_{TI}^{\left(2,1\right)}\varepsilon^{\frac{1}{3}}\left(\frac{a_{i2}^{\frac{5}{3}}}{\alpha_{2}^{\frac{2}{3}}}\right)\left(1-\alpha \right)exp\left(-\frac{We_{cr2}}{We_{2}}\right)\sqrt{1-\frac{We_{cr2}}{We_{2}}}\times \left(0.212D_{c2}^{*}{}^{\frac{13}{3}}-0.167D_{c2}^{*}{}^{5}\right)$ $\Phi_{TI,g2}^{\left(2\right)}=0.378C_{TI}^{\left(2\right)}\varepsilon^{\frac{1}{3}}\left(\frac{a_{i2}^{\frac{5}{3}}}{\alpha_{2}^{\frac{2}{3}}}\right)\left(1-\alpha \right)exp\left(-\frac{We_{cr2}}{We_{2}}\right)\sqrt{1-\frac{We_{cr2}}{We_{2}}}\times \left(1-0.212D_{c2}^{*}{}^{13/3}\right)$	$\Phi_{SO,1}^{\left(2,12\right)}=7.17C_{SO}\frac{\rho_{f}^{3/5}v_{r1}^{1/5}\sigma^{2/5}}{\rho_{g}D_{H}^{2/5}}\frac{a_{i2}^{2}}{\alpha_{2}}\left[1-{\left(\frac{We_{c,SO}}{We_{max2}}\right)}^{4}\right]$ $\Phi_{SO,2}^{\left(2,12\right)}=-0.36C_{SO}\left(\frac{\sigma }{\rho_{g}v_{g2}}\right)\frac{a_{i2}^{2}}{\alpha_{2}}\left[1-\frac{We_{c,SO}}{We_{max2}}\right]$	$\Phi_{SI}^{\left(2\right)}=2.616\times 10^{-4}C_{RC}^{\left(2\right)}\frac{\varepsilon^{\frac{1}{3}}\alpha_{2}^{2}}{D_{H}^{2}}{\left(\frac{\sigma }{g\Delta \rho }\right)}^{\frac{1}{6}}\times \left[1-\exp\left(-C_{RC2}\alpha_{2}^{\frac{1}{2}}\right)\right]+1.425\times 10^{-7}C_{WE}^{\left(2\right)}\left(1-\exp\left(-0.7\alpha_{2}\right)\right){\bar{u}}_{rw2}\alpha_{2}^{2}{\left(\frac{\sigma }{g\Delta \rho }\right)}^{-1}$
	Smith et al. (2012) [74,75]	102~152	0.5 MPa, $j_{f}$ = 0.05~1 m/s, $j_{g}$ = 0.05~8 m/s					
	Schlegel et al. (2015) [76]	304	0.1~0.3 MPa, $j_{f}$ = 2 m/s, $j_{g}$ = 11 m/s					

## Data Availability

Data are contained within the article and can be requested from the corresponding author.

## Nomenclature

A	area, $m^{2}$
$a_{i}$	interfacial area concentration (IAC), 1/m
C	coefficient
$C_{D}$	drag coefficient
$CD_{c1}^{*}{}^{2}$	inter-group transfer coefficient
Ca	Capillary number
$c_{\mathrm{bv}}\;$	$f_{\mathrm{bv}}^{2/3}+{\left(1-f_{\mathrm{bv}}\right)}^{2/3}-1$
$c_{1}$-$c_{23}$	adjustable parameters
D	bubble size, m
$D_{c}$	critical diameter for transition between bubble groups, m
$D_{\mathrm{cr},\mathrm{ph}}$	bubble critical size due to phase change, m
$D_{H}$	hydraulic diameter of the flow channel, m
$D_{\min,e}$	an arbitrarily defined minimum eddy size, m
$D_{\mathrm{sc}}$	surface equivalent diameter, m
$D_{\mathrm{sm}}$	Sauter mean diameter, m
$D_{1}$	diameter of bubble 1, m
$D_{2}$	diameter of bubble 2, m
d	parent bubble diameter, m
$d_{j}$	daughter bubble diameter, m
$d_{e}$	eddy size, m
E	kinetic energy, kg $m^{2}s^{2}$
F(α)	turbulence damping factor due to the presence of the gas phase
f	frequency, 1/s
$f_{\mathrm{bv}}$	volume fraction, $V_{i}/V_{j}$
G	gap in a rectangular/annular channel, m
g	gravitational acceleration, m/$s^{2}$
I	turbulent intensity
j	superficial velocity, m/s
K	constant
k	the ratio of the average turbulent eddy size to the bubble Sauter mean diameter
$\langle l_{w}\rangle$	average distance between bubbles, m
N	average number of eddies arrive at the surface of a drop in unit time
n	number density of bubbles or eddies, 1/$m^{3}$
R	source/sink rates per unit mixture volume, 1/$m^{3}s$
$R_{t}$	radius of the tube, m
r	bubble radius, m
S	source/sink rates per unit mixture volume, 1/$m^{6}s$
t	time, s
$u_{\mathrm{rel}}$	relative velocity between bubbles, m/s
$u_{\mathrm{rw}2}$	wake velocity of Group II bubbles, m/s
$u_{w12}$	wake velocity of Group I bubbles, m/s
V	volume, $m^{3}$
v	velocity, m/s
$V_{1}^{\mathrm{BOX}}$	volume influenced by a wake, $m^{3}$
$v_{i}$	interfacial velocity, m/s
$v_{pm}$	the average local particle velocity, m/s
We Weber number	
Greek symbols	
$\alpha ,{\;\alpha }_{\max}$	volume fraction, packing limit
Λ	factor considering the effect of the size ratio between bubbles and eddies
$\dot{\gamma }$	shear rate, 1/s
Υ	factor considering the effect of the bubble that reduces the free space for bubble movement and causes an increase in the collision frequency.
ε	energy dissipation rate, $m^{2}$/$s^{3}$
η	efficiency
ζ	rate of volume source per unit mixture volume, 1/s
Γ	mass generation rate, kg/$m^{3}$s
Φ	IAC source/sink rate, 1/ms
$\mu_{f}$	liquid viscosity, Pa·s
ν	kinetic viscosity, $m^{2}$/$s^{2}$
ρ	density, kg/$m^{3}$
Π	factor considering the ratio of the mean bubble distance to the turbulent path length
σ	surface tension, N/m
$\tau_{s}$	surface tension forces, N
$\tau_{v}$	viscous stress, N
ψ	factor depending on bubble shape
χ	inter-group transfer coefficient
Subscripts	
1	Group I
2	Group II
b	breakup
c	coalescence
coll	collision
cr	critical value
D	drag force
d	dispersed
e	eddy
f	liquid phase
g	gas phase
i	i-th component
j	j-th component
kin	kinetic
m	mixture value
max	maximum
max2	maximum in Group II
min	minimum
nuc	nucleation
ph	phase change
RC	random collision mechanism
SO	shearing-off mechanism
TI	turbulent impact mechanism
WE	wake entrainment mechanism
SI	surface instability mechanism
Superscripts	
* non-dimensional value	
(1)	interactions within Group I bubbles
(11, 2)	coalescence of a Group I bubble with another Group I bubble to generate a Group II bubble
(12, 2)	coalescence of a Group I bubble with a Group II bubble to generate a new Group II bubble
(2)	interactions within Group II bubbles
(2, 1)	Group I bubbles generated from breakup of a Group II bubble
(2, 2)	Group II bubbles generated from breakup of a Group II bubble
(2, 11)	breakup of a Group II bubble to generate two Group I bubbles
(2, 12)	breakup of a Group II bubble to generate a (or multiple) Group I bubble(s) and a Group II bubble
